# Comprehensive analysis of mesenchymal cells reveals a dysregulated TGF-**β**/WNT/HOXB7 axis in patients with myelofibrosis

**DOI:** 10.1172/jci.insight.173665

**Published:** 2024-12-06

**Authors:** Saravanan Ganesan, Sarah Awan-Toor, Fabien Guidez, Nabih Maslah, Rifkath Rahimy, Céline Aoun, Panhong Gou, Chloé Guiguen, Juliette Soret, Odonchimeg Ravdan, Valeria Bisio, Nicolas Dulphy, Camille Lobry, Marie-Hélène Schlageter, Michèle Souyri, Stéphane Giraudier, Jean-Jacques Kiladjian, Christine Chomienne, Bruno Cassinat

**Affiliations:** 1 INSERM UMRS 1131, Institut de Recherche Saint-Louis, Université Paris Cité, Paris, France.; 2 INSERM U1232/LNC, Team Epi2THM, Université Bourgogne Franche-Comté, Dijon, France.; 3 Service de Biologie Cellulaire, Hôpital Saint-Louis, AP-HP, Paris, France.; 4 Laboratoire de recherche en génétique et hématologie translationnelle, Institut Gonçalo Moniz, Salvador, Bahia, Brazil.; 5 INSERM CIC 1427, Université Paris Cité, Centre d’Investigations Cliniques, Hôpital Saint-Louis, AP-HP, Paris, France.; 6 INSERM UMRS 1160, Institut de Recherche Saint-Louis, Université Paris-Cité, Paris, France.; 7 Laboratoire d’Immunologie et d’Histocompatibilite, Hôpital Saint-Louis, AP-HP, Paris, France.; 8 INSERM U944, CNRS UMR7212, Institut de Recherche Saint-Louis, Université Paris-Cité, Paris, France.

**Keywords:** Hematology, Bone marrow, Fibrosis

## Abstract

Despite the advances in the understanding and treatment of myeloproliferative neoplasm (MPN), the disease remains incurable with the risk of evolution to acute myeloid leukemia or myelofibrosis (MF). Unfortunately, the evolution of the disease to MF remains poorly understood, impeding preventive and therapeutic options. Recent studies in solid tumor microenvironment and organ fibrosis have shed instrumental insights on their respective pathogenesis and drug resistance, yet such precise data are lacking in MPN. In this study, through a patient sample–driven transcriptomic and epigenetic description of the MF microenvironment landscape and cell-based analyses, we identify homeobox B7 (HOXB7) overexpression and more precisely a potentially novel TGF-β/WNT/HOXB7 pathway as associated to a pro-fibrotic and pro-osteoblastic biased differentiation of mesenchymal stromal cells (MSCs). Using gene-based and chemical inhibition of this pathway, we reversed the abnormal phenotype of MSCs from patients with MF, providing the MPN field a potentially novel target to prevent and manage evolution to MF.

## Abstract 2

We identify HOXB7 overexpression and a novel TGFbeta-Wnt-HOXB7 pathway as associated to a pro-fibrotic and pro-osteoblastic biased differentiation of mesenchymal stromal cells

## Graphical abstract

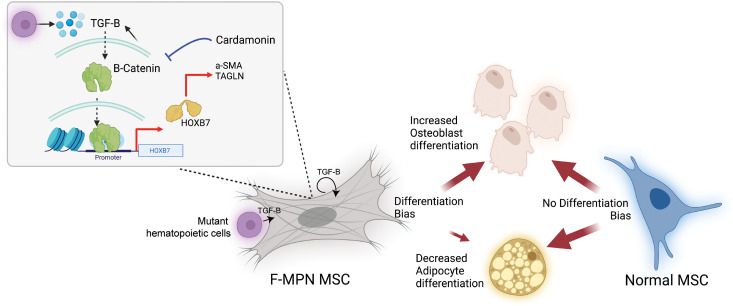

## Introduction

Myelofibrosis (MF) is a life-threatening complication of *BCR:ABL1*-negative myeloproliferative neoplasms (MPNs) induced by the MPN cell clonal proliferation, leading to bone marrow fibrosis, extramedullary hematopoiesis, and acute leukemia transformation (1). For patients with MPN, this results in extremely poor quality of life and overall shortened survival (2). The progression of MPN to MF is reported with a frequency that could be as high as 10% at 10 years and 20% at 20 years (3) with an overall short median survival of 6 years when MF is established (2). So far, the knowledge gained on the MPN hematopoietic clone at the genetic and phenotypic level has provided instrumental descriptive information for MPN management but has not changed the prevention or treatment of MF. Retrospective MPN patient studies reveal that patients with polycythemia vera with homozygous or with a high allele burden for JAK2^V617F^ mutations are more likely to progress to MF (4) while patients with post–essential thrombocythemia MF were more likely to have a high frequency of “nondriver” mutations affecting epigenetic regulation and the spliceosome machinery (ASXL1 and EZH2 mutations) (5). Recently, differential methylation of CpG sites of genes involved in cancer, embryonic development, inflammatory disease, or immunological diseases was identified in MF CD34^+^ cells (6). Unfortunately, no association between a single somatic gene mutation of the MPN clone and MPN evolution to MF was identified, spurring further studies to characterize alterations occurring in the microenvironment of the MPN clone.

Extensive studies in MF point to MPN mesenchymal stromal cells (MSCs) as key players. TGF-β has been shown for decades to have a direct role in fibrosis whether in MF or in other fibrotic diseases (1), and murine MF models show efficient targeting of the TGF-β receptor I (7) or TGF-β results in the reduction or inhibition of fibrosis (8). In patients with MPN, other cytokines such as IL-1β, IL-8, IL-2R, IL-6, TNF-α (9), CCL2 (10), lipocalin-2 (11), oncostatin-M (12), PDGF (13), FGF, and VEGF (14); inhibitors of metalloproteinase (15); and more recently IL-13 (16) have also been associated with the establishment of MF. An overall remodeling of the local hematopoietic niche by cytokine storms such as induced by IL-1β may also contribute to fibrosis (17). Recently, pro-fibrotic MSC subpopulations with increased expression of alarmins have been identified in patients with MPN who progress to fibrosis (18). Thus, clear evidence exists that during MPN evolution, both the MPN clone(s) and the MPN MSCs are subject to numerous external and internal altered signals. How these signals activate which pathways leading to fibrosis needs to be further unraveled.

To contribute to the ongoing research, we designed a study to comprehensively analyze the MSCs of patients with MF MPN. Leaning on a well-characterized MPN patient cohort both at the pathological and genomic level, we identify the TGF-β/WNT/homeobox B7 (HOXB7) axis in MF patients’ MSCs that is efficiently targeted in vitro.

## Results

### MSCs from patients with fibrotic MPN have distinct signatures and are biased toward osteoblast differentiation.

**Figure 1:**
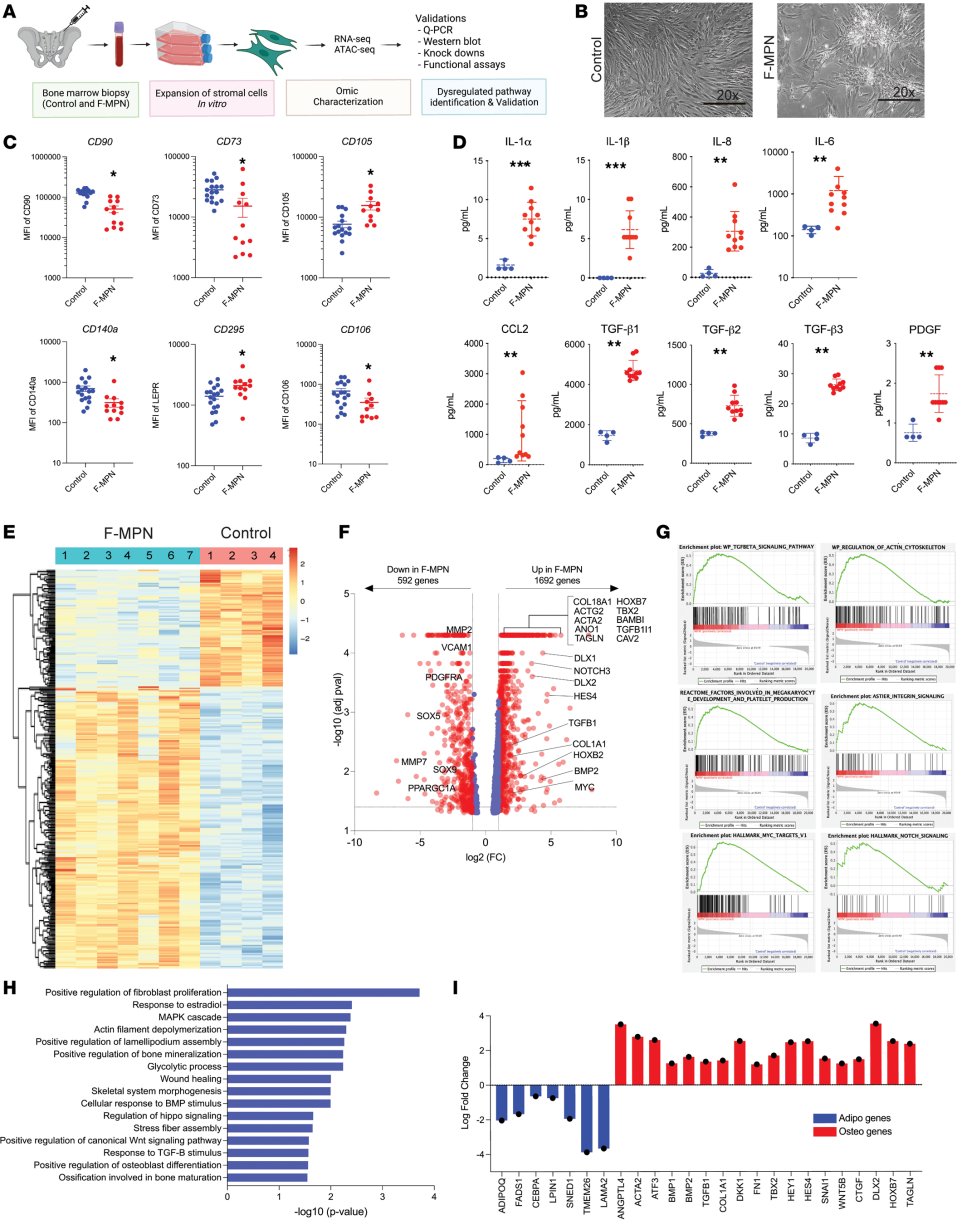
Expanded bone marrow MSCs from patients with MPN MF show an inflammatory profile and a biased osteoblast differentiation. (**A**) Experimental workflow of this study (illustration created using BioRender). ATAC-Seq, assay for transposase-accessible chromatin using sequencing. (**B**) Representative phase contrast images of expanded bone marrow MSCs from either patients with MPN MF patients or healthy age-matched controls. The spindle shape morphology of control MSCs (left) shifts to a stellar morphology in F-MPNs (right). Pictures were taken on passage 2. Scale bar = 20× original magnification. (**C**) Immunophenotypic characterization of expanded F-MPNs and control MSCs. All markers were present but differentially expressed in F-MPNs compared with controls. *P* values (*<0.05, **<0.01, ***<0.001) were calculated using unpaired *t* test. (**D**) Cytokine secretion profile of expanded MSCs. Day 3 supernatants at passage 2 were analyzed using a multiplexed Luminex assay. F-MPNs show a significant increase in inflammatory cytokine levels. *P* values (*<0.05, **<0.01, ***<0.001) were calculated using unpaired *t* test. (**E**) Heatmap and unsupervised hierarchical clustering by sample and gene were performed using the 300 genes (RNA-Seq data) that had the largest coefficients of variation based on DESeq2 analysis. The data are based on samples from the F-MPNs (*n* = 7) and control (*n* = 4) MSC group. (**F**) Volcano plot showing the relationship between the *P* values and the log_2_ fold-change in normalized expression (DESeq2) between F-MPNs (*n* = 7) and control MSCs (*n* = 4). Genes found to be the most differentially expressed are shown in the plot by *P* value. (**G**) Gene set enrichment analysis (GSEA) of RNA-Seq data between F-MPNs (*n* = 7) and control MSCs (*n* = 4) demonstrates an enrichment of gene sets of the fibrosis pathway. (**H**) Bar graph showing the pathways differentially expressed in the RNA-Seq analysis from control MSCs versus F-MPNs identified by DAVID pathway analysis. (**I**) Bar graph showing upregulation of osteoblast-associated genes and downregulation of adipocyte-associated genes in RNA-Seq data of F-MPNs.

While expanded MSCs from MPN patients meeting criteria of fibrosis (19), which will be referred to as fibrotic MPN MSCs (F-MPNs, *n* = 12) (Figure 1A and Supplemental Table 1; supplemental material available online with this article; https://doi.org/10.1172/jci.insight.173665DS1; and described in Methods), presented as stellar shaped cells in a clustered growth pattern, MSCs from age-matched controls (*n* = 17) showed a normal spindle shape (Figure 1B). The expansion and growth appeared slower in F-MPNs than in control MSCs albeit at a patient-dependent level (data not shown). F-MPNs expressed well-known cell surface MSC markers (CD105, CD90, CD73). However, their level of expression differed from control MSCs with an increased CD105 expression and a decreased CD90 and CD73 expression (Figure 1C). As reported previously (20), these F-MPNs also expressed high levels of the cell surface fibrosis marker LEPR (CD295), but a lower-than-reported expression of PDGFR-α (CD140a) (Figure 1C), which may indicate hyperstimulation of the PDGFR-α by PDGF. Indeed, analysis of the cytokine profile secreted from these expanded MSCs highlighted a pro-inflammatory profile for the F-MPNs as seen through an increased secretion of pro-inflammatory cytokines, such as IL-1α, IL-1β, IL-8, IL-6, CCL2, and PDGF. As expected, these F-MPNs secreted high levels of TGF-β1, TGF-β2, and TGF-β3, of which TGF-β1 is a known driver of fibrosis in MPN (1) (Figure 1D). None of the expanded F-MPNs (*n* = 12) expressed MPN driver mutations (Supplemental Table 1). RNA-Seq analysis of these F-MPNs (*n* = 7) equally underscored their distinct features compared with control MSCs (*n* = 4) at the transcriptional level (Figure 1E), with more than 1,600 differentially regulated genes (Figure 1F). F-MPNs’ gene expression profiles were enriched for fibrosis-associated genes (Figure 1, F and G), further validating the clinical and pathology selection criteria used for the MPN samples of this analysis (Supplemental Table 1). GSEA identified a dysregulation of fibrotic tissue pathways, such as the TGF-β pathway, cytoskeleton regulation, and NOTCH signaling (Figure 1G). Interestingly, we discovered a significant enrichment of WNT signaling and a disrupted MSC differentiation pattern, with an increase of osteoblast differentiation–associated genes and a decrease of adipocyte-associated genes, suggesting a differentiation bias of F-MPNs (Figure 1, H and I).

**Figure 2:**
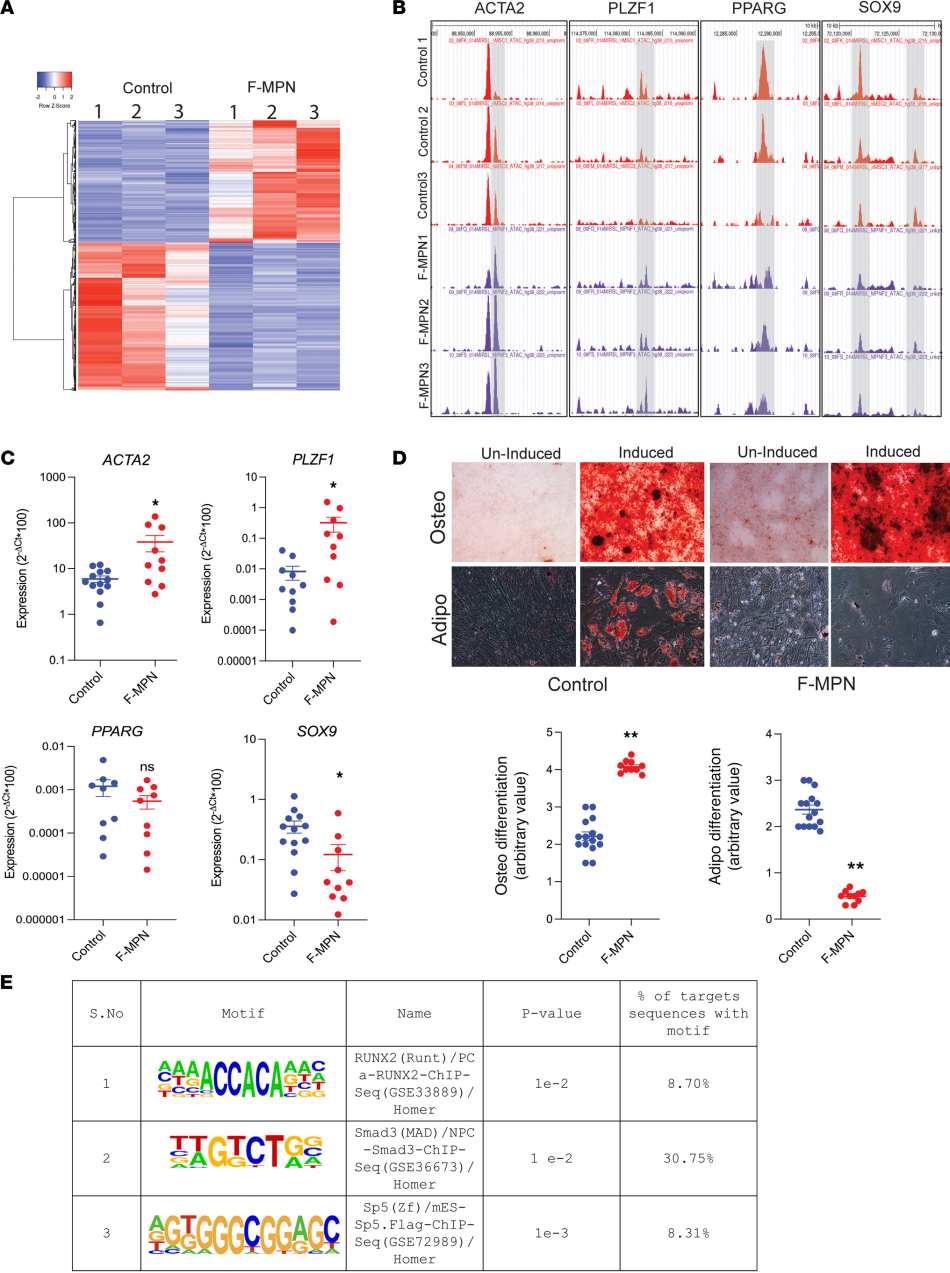
ATAC-Seq and in vitro differentiation assays verify the osteoblast bias of F-MPN MSCs. (**A**) Heatmap of ATAC-Seq peak intensity shows different accessible chromatin regions of the top 200 most variable genes (accessible chromatin — rows) across F-MPNs (*n* = 3) and control MSCs (*n* = 3) (columns). The color indicates scaled accessibility score. (**B**) Genome tracks around the loci of genes involved in fibrosis (*ACTA2*), osteoblast differentiation (*PLZF*), chondrocyte differentiation (*SOX9*), or adipocyte differentiation (*PPARG*) show an increased accessibility for *ACTA2* and *PLZF* genes and a decreased accessibility for *SOX9* and *PPARG* genes in F-MPNs. The genome tracks are from representative F-MPNs (*n* = 3) and control (*n* = 3) MSCs. (**C**) qRT-PCR validation of the ATAC-Seq and RNA-Seq findings in F-MPNs (*n* = 10) and control MSCs (*n* = 13). Both the fibrotic (*ACTA2*) and osteoblast markers (*PLZF*) are verified as upregulated in F-MPN samples while chondrocyte or adipocyte differentiation markers (*SOX9*, *PPARG*) are also verified as significantly decreased or at least show a trend toward significance. *P* values (*<0.05) were calculated using unpaired *t* test. (**D**) Representative differentiation induction profile of expanded MSCs (left panel: microscopy images after respective lineage staining for osteoblastic and adipocyte lineage Original magnification is ×10; right panel: arbitrary quantification of osteoblast and adipocyte differentiation). F-MPN MSCs show a biased differentiation profile toward the osteoblast lineage with a decrease in adipocyte lineage. **P value < 0.01 calculated using unpaired *t* test. Of note, F-MPN-1, 2, 3 and control 1, 2, 3 are from the same patients whom we analyzed in Figures 1 and 3. (**E**) HOMER DNA motif enrichment analyses of differentially accessible peaks (F-MPN versus control) show the enrichment of binding motifs for osteoblast differentiation transcription factors.

To further verify and decipher the dysregulated gene expression observed in F-MPNs, we first looked to chromatin accessibility in samples of the RNA-Seq cohort (3 each for F-MPNs and control MSCs). Based on differential peak calling (DESeq2 analysis), the F-MPNs showed a distinct chromatin accessibility profile from control age-matched MSCs (Figure 2A). A further analysis provided insights into the accessible transcription factor (TF) binding sites. The motif analysis (HOMER-based motif analysis) pointed to an enrichment of binding sites for TFs involved in fibrosis (Figure 2E). In line with the upregulation of the TGF-β pathway genes observed in these F-MPN samples, we noted an enrichment of SMAD3, RUNX2, and SP5 TF motifs (Figure 2E). Interestingly, these TFs are associated with osteoblast differentiation (21, 22). We also noted a significantly distinct enrichment of other osteoblast lineage TF motifs (23–26), such as FRA-1, ATF-3, FRA-2, AP-1, and TEAD4, in F-MPNs (Supplemental Figure 1). Integrating RNA-Seq data and ATAC-Seq data of genes involved in osteoblast lineage differentiation clearly identified an increased accessibility of the promoters of *ACTA2* (coding for α–smooth muscle actin, α-SMA) and *PLZF1* genes (Figure 2B). However, other osteoblast lineage differentiation genes, such as those highly expressed in the RNA-Seq study (Figure 1I) or reported in the literature, did not come out as significant at the time of analysis (27–30). More significantly, the C/EBPα TF motif involved in adipocyte differentiation (31) was reduced, corroborating the RNA-Seq results (Supplemental Figures 2 and 3) and in line with the significantly reduced chromatin accessibility for a C/EBPα target gene, PPARγ, a master gene of adipocyte differentiation (32). These omics data of the F-MPNs’ differentiation capacity were validated by quantitative reverse transcription PCR (qRT-PCR) on a larger cohort of F-MPN and control samples including samples used for RNA-Seq and ATAC-Seq (*n* = 10 and 12 F-MPN and control MSC samples), which showed an increased expression of osteoblast genes (*ACTA2*, *PLZF1*) and decreased expression of adipocyte gene (*PPARγ*) and chondrocyte gene (*SOX9*; Figure 2C). Finally, to verify these molecular findings, we performed a functional differentiation assay of F-MPNs (*n* = 12) and control MSCs (*n* = 10). After 21 days of culture and using lineage-specific staining (alizarin red and Oil Red O, respectively), F-MPNs showed an increased osteodifferentiation and reduced adipocyte differentiation (Figure 2D). Overall, the findings of this integrated transcriptome and epigenome analysis in patients with MF MPN point to an upregulation of osteoblast differentiation and fibrosis genes in the MSCs of MPN patients with fibrosis. These results are in line with previous studies (18, 33) and validate this cohort for the following studies of MF patient MSCs continued below.

### TGF-β–induced HOXB7 expression promotes fibrosis and osteoblast differentiation in F-MPNs.

**Figure 3:**
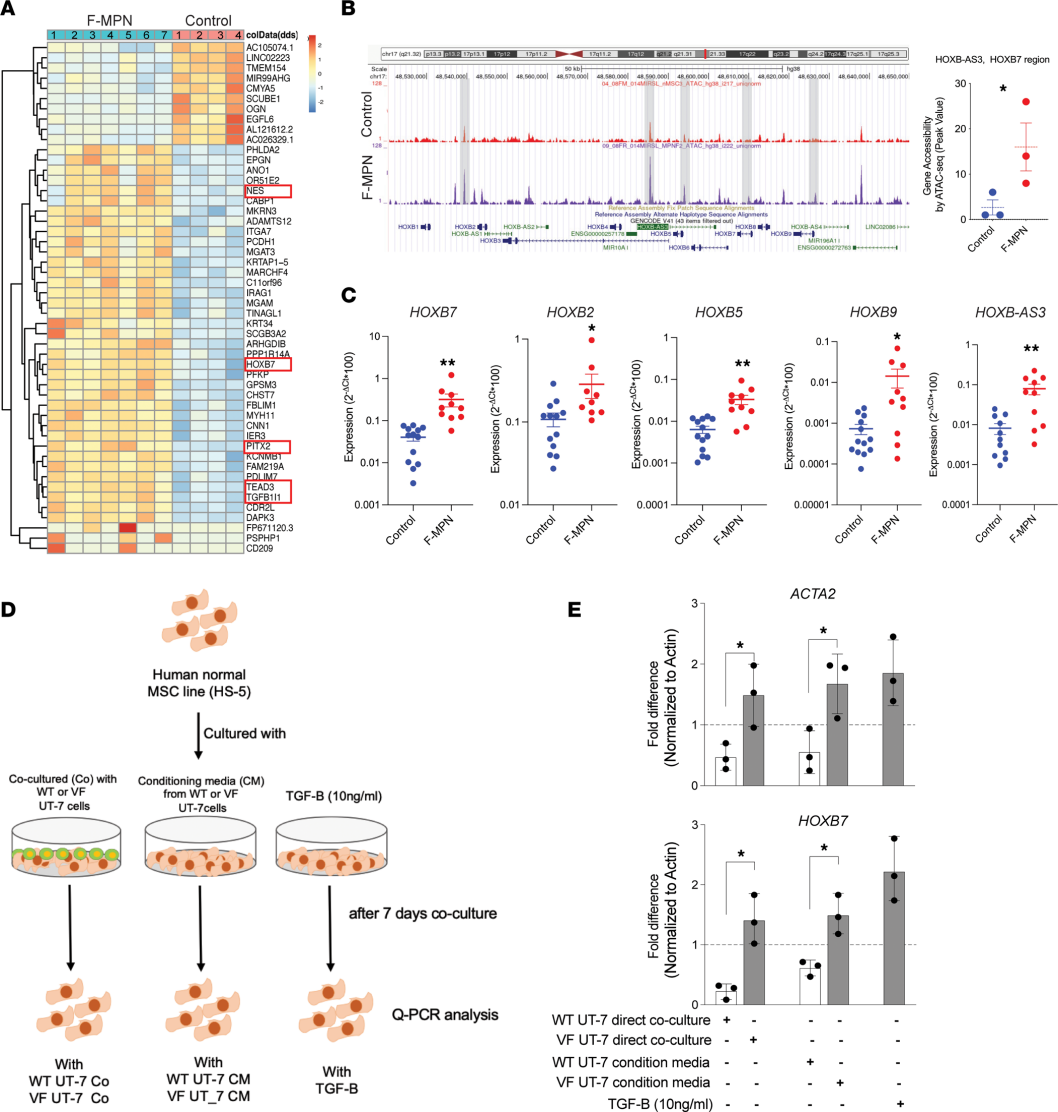
ATAC-Seq, RNA-Seq, and coculture studies highlight *HOXB7* upregulation in F-MPN samples. (**A**) Heatmap showing the top 50 differentially regulated genes from RNA-Seq data. Unsupervised hierarchical clustering by sample and gene were performed. The data are based on samples from the F-MPN (*n* = 7) and control (*n* = 4) MSC groups. In the top 50 genes, boxed genes are associated with either fibrosis or osteoblast differentiation pathways. (**B**) Gene accessibility tracks of *HOXB* genes (ATAC-Seq) show a statistically significant increased accessibility in F-MPNs (example of the *HOXB-AS3-HOXB7* region is quantified in the right panel). *P* value *< 0.02 calculated using unpaired *t* test. (**C**) qRT-PCR validation of the ATAC-Seq and RNA-Seq findings in control (*n* = 13) and F-MPN (*n* = 10) samples. Several *HOXB* genes are upregulated in F-MPN MSCs. *P* values (*<0.05, **<0.01) were calculated using unpaired *t* test. (**D**) In vitro coculture of normal human mesenchymal cells from the HS-5 cell line with either cells or conditioned media from human hematopoietic cell line UT-7 bearing either the wild-type *JAK2* gene (WT) or the mutated *JAK2* *V617F* gene (VF) or with TGF-β (10 ng/mL). (**E**) After 7 days’ coculture, an increased expression of *HOXB7* and *ACTA2* genes is noted in the MSCs after exposure to TGF-β or *JAK2*-mutated cells (VF). *P* values (*<0.05) were calculated using unpaired *t* test.

The *HOXB7* gene was one of the most significantly dysregulated genes in the top 50 dysregulated genes of the F-MPN RNA-Seq study (Figure 3A). To our knowledge, this is the first time that HOXB7 has been linked to MPN MSCs at any stage of the disease. ATAC-Seq data corroborated this finding and showed that, in F-MPNs, there is an increased chromatin accessibility at the HOXB gene cluster and in particular at the *HOXB7* gene region when compared with age-matched control MSCs (Figure 3B). qRT-PCRs of the *HOXB7* gene and other HOXB genes (*HOXB2*, *HOXB5*, and *HOXB9*) verified the dysregulation of a HOXB pathway in these F-MPNs (Figure 3C).

Of interest for this study, *HOXB7* has already been related to the differentiation of stromal cells toward the osteoblast lineage (34). We thus questioned whether the increased expression of HOXB genes noted in the MSCs of these patients with fibrotic MPN, which also present with a biased differentiation toward osteoblast differentiation (RNA-Seq data), could be induced by the malignant MPN clone. To answer this question, we established an in vitro coculture system where a normal human MSC cell line (HS-5) was cocultured with an MPN human cell line UT7, bearing either a JAK2^V617F^ mutant (MUT) or a wild-type (WT) *JAK2* gene (Figure 3D). After 7 days of coculture, an upregulation of the *HOXB7* gene as well as the *ACTA2* gene (fibrotic gene) was observed in HS-5 cells cocultured with the JAK2^V617F^ mutant cell line (Figure 3E). A similar differential expression was noted when HS-5 cells were cultured with conditioned media from the WT or MUT cells (Figure 3E). Incubation of the HS-5 cells with 10 ng/mL TGF-β equally induced an upregulation of *HOXB7* and *ACTA2* genes (Figure 3E). In summary, this analysis shows that in F-MPNs, *HOXB7* and fibrosis-associated genes are dysregulated and that the MPN *JAK2*-mutated clone has the potential to modify their expression in normal MSCs via their secretome.

**Figure 4:**
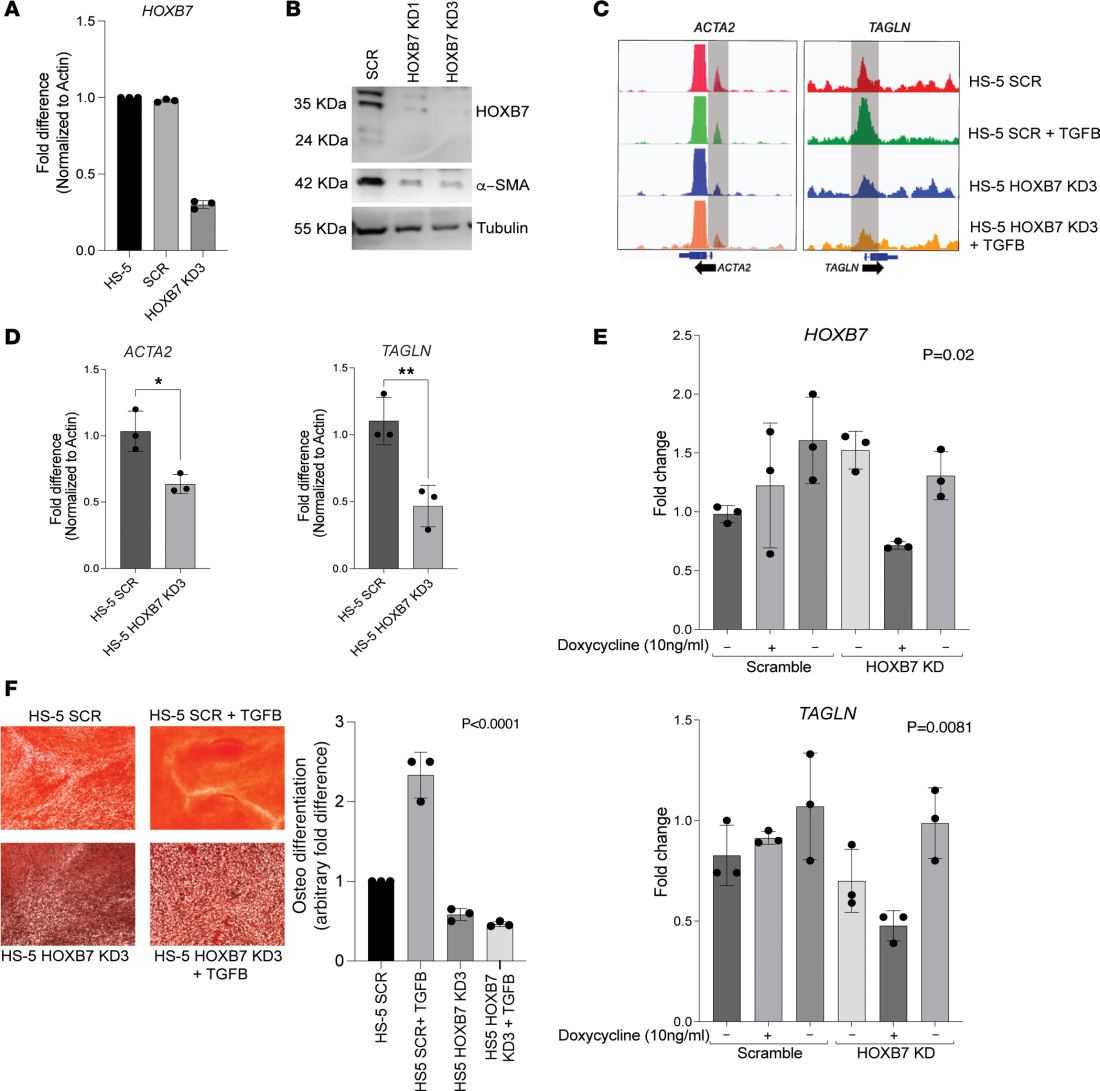
Knockdown of *HOXB7* gene alters the potential of MSCs for osteodifferentiation. (**A**) Knockdown of *HOXB7* by shRNA in the HS-5 mesenchymal cell line. The reduced expression of *HOXB7* is validated in an shRNA-transfected cell line by qRT-PCR. (**B**) Knockdown of *HOXB7* by shRNA in the HS-5 mesenchymal cell line. Western blot showing absence of HOXB7 in 2 shRNA-transfected cell lines results in downregulation of the downstream target α-SMA (protein encoded by the *ACTA2* gene). (**C**) Gene accessibility tracks of 2 osteoblast-associated genes, *ACTA2* and *TAGLN*, by ATAC-Seq. HS-5 cells with reduced expression of *HOXB7* (HOXB7-KD3) show reduced chromatin accessibility of the *ACTA2* and *TAGLN* genes even after incubation with TGF-β (10 ng/mL for 3 days). (**D**) qRT-PCR validation of the ATAC-Seq findings showing significantly reduced expression of the *ACTA2* and *TAGLN* genes in the *HOXB7*-KD3 cells in the presence of TGF-β. *P* values *<0.05, **<0.02 calculated using unpaired *t* test. (**E**) qRT-PCR results showing the downregulation of *HOXB7* and *TAGLN* even in the presence of TGF-β upon doxycycline (Dox) addition, and the same is restored when the Dox is withdrawn from the same cells after 3 days (*n* = 3). (**F**) Osteoblast differentiation induction assay of HS-5 cells after transfection with either scramble (SCR) or *HOXB7* shRNA (*HOXB7* KD3) alone or after treatment with TGF-β for 21 days. (Left panel: microscopy images after osteoblast lineage staining; original magnification is ×10; right panel: arbitrary quantification of osteoblast differentiation.) Absence of *HOXB7* reduces osteoblast differentiation even in the presence of TGF-β. *P* values were calculated using 1-way ANOVA test.

To further establish the relationship between *HOXB7* and osteoblast differentiation of MPN MSCs, we generated a stable *HOXB7*-knock-down (*HOXB7*-KD) HS-5 cell line using lentiviral shRNA (Figure 4, A and B, and Supplemental Figure 4). HS-5 cells that do not express HOXB7 (HS5-HOXB7-KD3) (Figure 4A) showed clearly reduced chromatin accessibility and reduced gene expression for *ACTA2* and *TAGLN* genes — genes involved in fibrosis and osteoblast differentiation (Figure 4, C and D). A Western blot verified the decreased protein expression for *ACTA2* (Figure 4B). To establish the fact that HOXB7 is regulated by TGF-β and influences the expression of downstream genes such as *TAGLN*, we made a doxycycline-inducible knockdown of HOXB7 where we showed that even in the presence of TGF-β, induction of the HOXB7 knockdown reduced the expression of *TAGLN* (Figure 4E) while restoration of HOXB7 by doxycycline withdrawal triggered a recovery of *TAGLN* expression (Figure 4E). Importantly, we also observed that in the absence of *HOXB7* gene, the differentiation profile of HS-5 MSCs showed a significant reduction in osteoblastic lineage even in the presence of TGF-β (Figure 4F). These results support the role of TGF-β–induced HOXB7 expression as a master regulator for both fibrosis and osteoblast differentiation in human MSCs from patients with MPN.

### Targeting WNT signaling counteracts the TGF-β/HOXB7–driven pro-fibrotic and pro-osteoblastic fate of MSCs.

**Figure 5:**
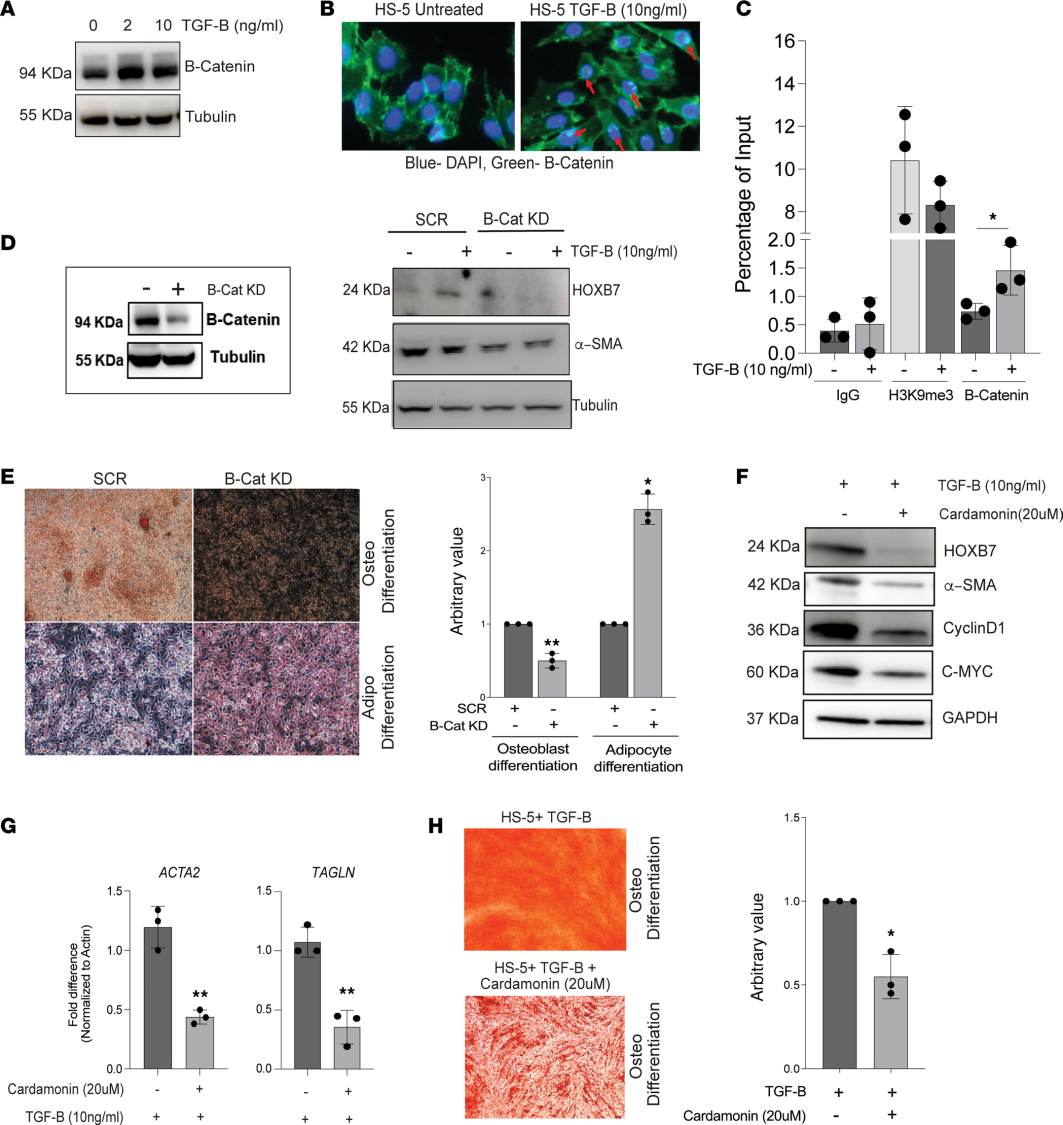
TGF-β–induced WNT signaling drives the HOXB7-mediated pro-osteogenic profile of MSCs. (**A**) Treatment of normal human mesenchymal cells (HS-5) with TGF-β (2 and 10 ng/mL) stabilizes β-catenin levels (immunoblot). (**B**) Treatment of normal human mesenchymal cells (HS-5) with TGF-β (10 ng/mL) induces translocation of β-catenin to the nucleus (red arrows). Microscopy images after staining with mAb-β-catenin (green) and DAPI (blue nucleus) (representative of 3 experiments). Original magnification, ×100. (**C**) ChIP assay of the *HOXB7* promoter. Incubation with TGF-β (10 ng/mL) drives increased chromatin accessibility (reduced H3K9me3 marks) and binding of β-catenin (*n* = 3) to the *HOXB7* promoter. *P* value *<0.05, unpaired *t* test. (**D**) Knockdown of β-catenin in HS-5 cell line by a β-catenin shRNA (β-Cat KD), validated by Western blot (right), induces reduced expression of HOXB7 and its downstream target α-SMA (protein encoded by *ACTA2*) in the absence or presence of TGF-β. (**E**) Osteoblast and adipocyte differentiation induction assay of normal mesenchymal HS-5 cells after transfection with scramble (SCR) or β-catenin shRNA (β-Cat KD). Microscopy images after respective lineage staining show that absence of β-catenin decreases osteoblast differentiation and increases adipocyte differentiation. Original magnification, ×10. (**F**) Pretreatment of HS-5 cells with WNT inhibitor (cardamonin 20 μM) followed by treatment with TGF-β reduces the expression of HOXB7 and α-SMA along with other WNT pathway genes such as c-MYC and Cyclin-D1 (*n* = 3). (**G**) Treatment of HS-5 cells with TGF-β and WNT inhibitor reduces the expression of *ACTA2* and *TAGLN* genes validated by qRT-PCR (*n* = 3). *P* value **<0.02, unpaired *t* test. (**H**) Osteodifferentiation induction of HS-5 cells shows a decreased osteoblast differentiation profile upon treatment by cardamonin (*n* = 3). Original magnification, ×10. *P* value *<0.05, unpaired *t* test.

**Figure 6:**
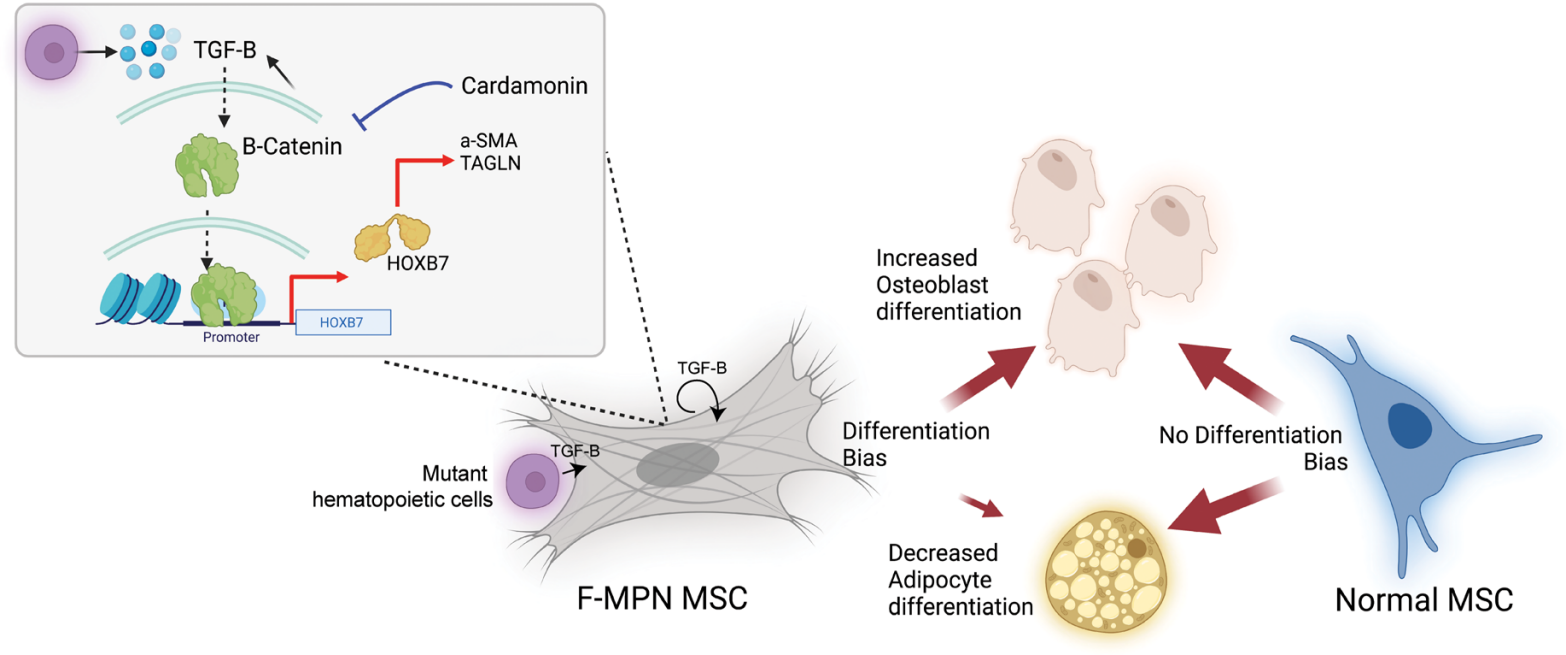
Graphical diagram describing the role of the WNT/TGF-β/HOXB7 axis in MPN patients’ MSC deregulation. Illustration created using BioRender.com.

Because a WNT signature was noted in F-MPN RNA-Seq and ATAC-Seq studies (SP5 motif enrichment) (Figure 1H and Figure 2E), we hypothesized that a WNT signaling pathway could regulate HOXB7 expression downstream of TGF-β signaling. Toward this, we analyzed canonical WNT signaling features in HS-5 cells treated with TGF-β. An increased stability of β-catenin, its translocation into the nucleus (Figure 5, A and B), and its binding on the promoter of *HOXB7* gene along with a reduced H3K9 trimethylation (H3K9me3) mark were noted upon TGF-β treatment (Figure 5C). Furthermore, absence of β-catenin (β-catenin–KD HS-5 cell line) led to a reduced expression of not only HOXB7 protein but also the fibrosis-associated α-SMA protein (product of the *ACTA2* gene) (Figure 5D). A differentiation assay further showed a decreased osteoblast differentiation and increased adipocyte differentiation of the β-catenin–KD HS-5 cell line (Figure 5E). Together, these data show that in MSCs, WNT signaling, when activated by TGF-β, controls the expression of HOXB7, leading to a bias toward osteoblast differentiation and possibly to fibrosis as suggested by the increased ACTA2/α-SMA expression. As lentivirally triggered absence of β-catenin restored the differentiation bias, we next tested whether chemical inhibitors of WNT signaling could target this novel axis of HOXB7 activation in MSCs. Treatment of HS-5 cells with an inhibitor of WNT signaling (cardamonin — 20 μM) was effective to decrease protein levels of WNT targets (Cyclin D1 and C-MYC) but also to reduce TGF-β–induced *HOXB7* expression (Figure 5F). Of note, the expression of HOXB7 was not modified by the JAK1/2 inhibitor ruxolitinib frequently used to treat MPN (Supplemental Figure 5). The inhibition of WNT signaling equally decreased the expression of osteoblast- and fibrosis-associated genes (Figure 5G). Finally, in the differentiation assay, treatment of HS-5 cells with cardamonin corrected the differentiation bias toward osteoblasts (Figure 5H). Thus, our work identifies a TGF-β/β-catenin/HOXB7 axis, which plays an important role in pro-fibrotic features in MPN (Figure 6).

## Discussion

Though an increased understanding of MPN onset has been gathered in the recent years since the identification of the *JAK2* (and others) mutations, scarce input has been gathered from the few mouse models and patient studies to gain further insights into the evolution of the disease to MF. All studies converge to support the role of patient MSCs and their modification by the MPN hematopoietic clone to trigger MF. The cytokine release by the MPN clone remains the main constant triggering mechanism where TGF-β is the main player. Fine-tuning of the various MSCs populations, cytokines, and adhesion molecules continues to complete the MPN fibrosis landscape. However, few descriptions of the genetic and epigenetic alterations of MF MPN MSCs are available, maybe due to the difficulties of cell sampling for these studies whether in MF patients or mouse models, impeding the discovery of preventive or therapeutic options.

To fill this gap of knowledge, we leaned on a substantial well-established and -characterized MPN MF patient cohort of our center and designed an approved study to perform biopsies in patients with MPN MF to comprehensively analyze the in vitro–expanded fibrotic MSCs from MPN using multiomics techniques. The data from the analysis of this cohort of MPN MF patients’ MSCs showed all the characteristics previously reported for MPN fibrosis at the molecular and gene expression level, whether in cell surface markers, cytokine secretion, biased differentiation toward the osteoblast lineage, or fibrosis gene expression profile including WNT-altered pathways (1, 18, 20, 33). The role of WNT signaling in MSC osteoblast lineage commitment is very well understood (35, 36), as is its crosstalk with other differentiation pathways (37). However, how these pathways act together to induce pathologic differentiation is not clearly understood.

This unique well-characterized and validated MPN MF patient MSC cohort gives strong credit to the identification of a potentially novel candidate pathway, the HOXB cluster, in the top 50 dysregulated genes. ATAC-Seq data verified a substantial accessibility in the *HOXB* family of genes, including *HOXB7* and its close neighbor *HOXB9*, but also *HOXB2*, *HOXB5*, and *HOXB-AS3*. The role of HOXB in skeletal regeneration and MSC differentiation is known (38, 39). Interestingly, the most highly expressed gene in this study is the *HOXB7* gene, equally known in the field of MSCs and osteoblast differentiation but never identified until now in the field of MPN. The role of *HOXB7* in MPN MSC osteoblast differentiation was verified, when inhibition of *HOXB7* expression and not *HOXB9* expression in MSC-induced differentiation decreased osteoblast differentiation. The decreased osteoblast differentiation persisted even in the presence of TGF-β in line with the MSC HOXB code (40). Leaning on the robust coculture system of normal MSC cell line with a mutant JAK2 human MPN cell line, we established that the *HOXB7* gene was controlled by the interaction of the MSC with the mutant MPN clone. This control was further shown to be mediated by the malignant clone’s secretome and TGF-β, known as the major player in the secretome. We also noted that the expanded fibrotic MSCs produced inflammatory cytokines, including TGF-β, suggesting that fibrotic MSCs are also capable of inducing autocrine TGF-β signaling. These results clearly substantiate the role of the interaction with the MPN clone and/or autocrine TGF-β in the upregulation of HOXB7 in MPN MSCs and the subsequent biased differentiation toward the osteoblast lineage.

As both TGF-β and WNT signaling pathways were dysregulated in the MPN MSC RNA-Seq and ATAC-Seq data, we designed in vitro studies to unravel the molecular actors of TGF-β/WNT signaling in MPN MSCs. We highlighted an activation of the canonical WNT pathway upon TGF-β treatment of MSCs and most interestingly a binding of β-catenin to the promoter of *HOXB7*. Such a crosstalk between TGF-β and WNT signaling has previously been reported (41), but for the first time to our knowledge, we identify that this signaling is necessary for *HOXB7* activation. We further verified the existence of this TGF-β/WNT/HOXB7 axis when a reduced osteoblast differentiation and *ACTA2* gene expression resulted from the inhibition of β-catenin.

Thus, in MPN MSCs, there exists a crosstalk between TGF-β/WNT and HOXB7 that induces osteoblast differentiation. Although one study on patients with primary and secondary MF highlighted an increase in β-catenin mRNA in the bone marrow mononuclear cells (42), to our knowledge, WNT/β-catenin upregulation in F-MPN MSCs has never been previously reported. These data also provide further impact on the already well-described role of TGF-β in fibrosis and in MPN fibrosis. We verify that TGF-β was associated with the hematopoietic mutant clone of MPN, as coculture studies with cell-cell contacts or mutant clone conditioned media produced similar results as incubation with TGF-β alone. However, we cannot eliminate the possibility of autocrine TGF-β signaling in a fibrotic setting. These data stress once more the role of the mutated clone in mediating MPN progression to MF, whether directly or by modification of the MPN MSC and microenvironment, data further corroborated by the fact that the MSCs of this study did not carry any known MPN driver mutation. Other mechanisms may occur in vivo as noted in a mouse model in which neuropathy may induce MSC alterations, leading to fibrosis in MPN (17), in vivo results partially confirmed in a clinical trial (43).

In summary, we demonstrate that the biased osteoblast differentiation of fibrotic MSCs is mediated by the MPN hematopoietic mutated clone and its secretion of TGF-β. Our study attributes this bias to the dysregulation of the MSCs’ *HOXB7* gene by a TGF-β–induced WNT signaling. Thus, a deregulated TGF-β/WNT/HOXB7 axis pathway is identified in MPN-MF evolution, offering a potential monitoring and therapeutic target for MPN patients with MF.

## Methods

### Sex as a biological variable.

In this study both male and female patients were included. Sex of patients was not considered as a biological variable.

### Human MPN primary cells and cell lines.

Bone marrow samples and biopsies from patients with MPN were collected for diagnostic purposes, and MSCs were isolated after obtaining written informed consent. The human UT-7 megakaryoblastic cell line lentivirally transduced with WT JAK2 or JAK2^V617F^ mutated gene, HS-5 cell line (obtained from ATCC), and HS-5 β-catenin–knockdown line (gift from Vikram Mathews, Christian Medical College Vellore, Vellore, India) were also used in this study.

### MSC expansion and differentiation.

F-MPN MSCs and normal MSCs were expanded from the bone marrow biopsies from patients with MPN and age-matched control patients who underwent hip replacement surgery, respectively. MSCs were cultured using MEM α (Life Technologies) with 10% fetal bovine serum (FBS; Life Technologies), 100 U/mL penicillin and 100 μg/mL streptomycin, l-glutamine, and β-mercaptoethanol (Life Technologies) in a humidified atmosphere with 5% CO_2_. Medium was changed every 3 days until the stroma was expanded. Stromal cells from passage 2 or 3 were used for the study. For osteoblast and adipocyte differentiation, the MSCs from passage 2 were used. The medium for osteoblast differentiation was MEM α supplemented with 10% FBS, pen-strep (1%), l-glutamine (1%), dexamethasone (0.1 μM), β-glycerophosphate (10 mM), and ascorbic acid (50 μM). The medium for adipocyte differentiation was low-glucose DMEM (Life Technologies) with 10% FBS, pen-strep, l-glutamine, 3-isobutyl-1-methylxanthine (0.5 mM), indomethacin (60 μM), insulin (100 ng/mL), and hydroxycortisone (0.5 μM). In either osteoblast or adipocyte differentiation assays, cells were cultured for 21 days with replacement of differentiation media every 3 days. The osteoblast and adipocyte differentiations were confirmed using alizarin red S staining and Oil Red O staining, respectively.

### Cytokines and inhibitors.

Cardamonin was purchased from Merck, TGF-1 from Bio-Techne, and ruxolitinib from MedChemExpress. All molecules were reconstituted and stored according to the suppliers’ instructions.

### Lentivirus-mediated knockdown.

The shRNAs for scramble and *HOXB7* were purchased from MilliporeSigma (TRC cloning vectors). The plasmids were amplified, and lentiviral particles were generated according to standard protocols. The efficacy of transduction was measured through GFP-positive cells, which acted as a positive control. The lentiviral particles generated were used to transduce HS-5 cells for 6 hours, and the medium was changed, allowing the cells to grow in complete media for 24 hours. The transduced cells were selected with puromycin (1 mg/mL) for 2 weeks, and the knockdown was confirmed by Western blot assays.

### qRT-PCR.

Total RNA was extracted using TRIzol reagent (Invitrogen). A total of 500 ng of the extracted RNA was converted into cDNA using a superscript III cDNA kit (Invitrogen). The expression of genes was studied using the SYBR green method (Applied Biosystems) (primer sequences used are given in Supplemental Table 2). The Ct values were normalized with *ACTB*, and the fold differences were calculated using the 2^-ΔCt^ method or 2^-ΔΔCt^.

### RNA-Seq experiments.

RNA-Seq was performed at Plateforme de Génomique Institut Cochin Inserm 1016-CNRS, Paris, France. Total RNA libraries were prepared using the Illumina TruSeq RNA kit as described by the manufacturer. Total RNA, 1 microgram, was used from each sample for the library preparation. The 5′ and 3′ RNA adapters were ligated to the RNA, and the ligated products were reverse-transcribed using superscript II reverse transcriptase (Invitrogen). The size and integrity of each library were verified using the Bioanalyzer (Agilent). The libraries were sequenced on a HiSeq 1000 instrument (Illumina). The differentially expressed RNA was analyzed using DESeq2 with FDR-corrected *P* < 0.05. Briefly, reference-based de novo transcriptome assembly was performed using Cufflinks (version 0.9.3) and Scripture. RefSeq- and Ensembl-annotated transcripts were filtered out from Scripture- and Cufflinks-assembled transcriptomes. De novo transcript assembly was processed through Trinity, and the Coding Potential Calculator scores were determined. To determine the number of statistically significant differentially expressed RNAs for hierarchical clustering, SAM-Seq was performed, and significant transcripts with FDR < 0.05 were identified.

### ATAC-Seq experiment.

The ATAC-Seq on MSCs was carried out by Active Motif Inc. In short, 50,000 viable nuclei were tagmented using Tn5 enzyme preloaded with next-generation sequencing adapters, followed by amplification of tagmented DNA using standard primers from the kit (Active Motif Inc.). The amplified libraries were subjected to sequencing using HiSeq 500. The paired-end 42 bp sequencing reads (PE42) generated were mapped to the genome using the Burrows-Wheeler Aligner algorithm. Genomic regions with high levels of transposition/tagging events were determined using the MACS2 peak-calling algorithm, and the fragment density was determined followed by normalization of the reads. After identifying merged regions as part of the standard analysis pipeline, the DESeq2 software was run on the unnormalized BAM files (without duplicates). We then performed HOMER motif analysis (findMotifsGenome.pl) on the 200 bp sequence centered on the midpoint of the differential region (+100 bp, –100 bp) to identify TF motifs.

### Immunoblots.

HS-5 cells were harvested and the pellets were lysed in RIPA buffer (MilliporeSigma) for 30 minutes in ice, with complete protease inhibitors (Roche). The lysates were collected by centrifugation at 20,000*g* for 10 minutes. The lysates were analyzed in SDS-PAGE. After protein transfer to a nitrocellulose membrane (Bio-Rad), membranes were blocked with nonfat dry milk (5%, 2 hours at room temperature) followed by incubation with primary antibodies overnight at 4°C (Supplemental Figure 6). The protein bands were detected by the standard chemiluminescence method (Thermo Fisher Scientific). For imaging, an Amersham ImageQuant system (Cytiva) was used.

### ChIP assay.

ChIP assay was performed through standard kit protocol (Digenode). Antibodies were against β-catenin (Santa Cruz Biotechnology clone 12F7) and H3K9me3 (Digenode catalog C15410193). The antibody-bound DNA was analyzed using qPCR using the following primers for HOXB7 promoters: forward (5′–3′) GGGAATCACGTGCTTTTGTT and reverse (5′–3′) TGTTTCTCCCCCTTCTCCTT.

### Generation of Tet-inducible HOXB7-KD clone.

Doxycycline-inducible HOXB7 shRNA was generated using Tet-pLKO-puro plasmid (Addgene plasmid 21915; https://www.addgene.org/21915/). The HOXB7 shRNA sequences (KD3 & KD5) from previous validated experiments were cloned according to previous reports (44). Precloned scramble shRNA (Tet-pLKO-puro-Scrambled; Addgene plasmid 47541; https://www.addgene.org/47541/), was used as control for the experiment. The cloned shRNAs in plasmids were confirmed and were expanded in Stbl3 strain of *E*. *coli* according to the supplier’s instructions (Invitrogen). For packaging the plasmids, Pax2 and VSVG plasmids (Addgene) were used according to a standard protocol. HEK293T cells (ATCC) were infected in the presence of 5 μg/mL polybrene, and the supernatant containing viral particles was collected at 48 hours. It was filtered and concentrated overnight at 4°C using Retro-X virus concentrator (Takara Bio). The concentrated virus was spun and stored at –80°C. The virus was titrated on HS-5 cell line by overnight transduction. The next day, the medium was changed, and puromycin selection (starting from 1 μg/mL and increasing to 3 μg/mL maximum) was started 2 days after transduction. After 1 week, the cells resistant to puromycin were dissociated and counted to determine the efficiency of transduction. The viral titer was determined and used for transducing HS-5 cells with Tet-inducible scrambled and HOXB7-specific plasmids. After transduction, cells were cultured in medium containing tetracycline-free FBS (Takara Bio). Transduced cells were selected as already described. For inducing the shRNA, doxycycline (MilliporeSigma) was used. Doses up to 100 ng/mL were tested and validated by qRT-PCR. For experiments, a dose of 10 ng/mL was validated and used for further experiments. For experiments, the cells after exposure to doxycycline (10 ng/mL; 3 days) were washed in PBS and reintroduced with fresh media for another 3 days without doxycycline. RNA samples were extracted at day 0 (pre dox), day 3 (post dox) and day 6 (dox withdrawal), and the expression of HOXB7 and TAGLN was analyzed as described above.

### Cytokine measurement.

MSCs were seeded at a density of 10^6^ per 75 cm^2^ flask. Next day, the medium was changed to MEM α containing 2% FBS. After 48 hours, the supernatant was spun at 1,500*g* and filtered using a 0.2 μm filter, then stored at –80°C. A custom Luminex assay was purchased from R&D Systems, Bio-Techne, with the following cytokines: CCL2; HGF; IGFBP-2; IL-1α; IL-1β; IL-6; IL-8; IL-15; leptin; lipocalin-2; PDGF isoforms AA, BB, CC, and DD; thrombopoietin; and VEGF. For the assay, the manufacturer’s instructions were followed, and reading was done on a Bio-Rad MAGPIX analyzer. For measuring TGF-β1, 2, and 3, the TGF-β 3-plex assay was purchased from Bio-Rad. As per the manufacturer’s instructions, the samples were activated before incubation with the magnetic beads. A 2-tailed *t* test was used for statistical analysis.

### Statistics.

Statistical analyses for evaluating differences between 2 groups were performed using the unpaired and 2-tailed *t* test. For evaluating significance in more than 2 groups, 1-way ANOVA was used. GraphPad Prism 9 was used for these statistical analyses. *P* < 0.05 was considered statistically significant

### Study approval.

The study was approved by the institutional review board Comité d’Evaluation de l’Ethique des Projets de Recherche Biomédicale du Groupe Hospitalier Universitaire Nord de l’Assistance Publique-Hôpitaux de Paris, Paris, France (IRB00006477, CER-2020-55).

### Data availability.

The RNA-Seq and ATAC-Seq processed data have been deposited in NCBI Gene Expression Omnibus (GSE234388, GSE234389, GSE234390). Supporting Data Values are available in the supplement.

## Author contributions

S Ganesan, S Giraudier, JJK, CC, and BC conceived and designed the study. S Ganesan performed most of the experiments, analyzed the data, and wrote the manuscript. SAT designed and performed several experiments (cytokine measurements, cell culture experiments, inhibitor testing), analyzed data, and edited the manuscript. FG, NM, and CL analyzed RNA-Seq and ATAC-Seq experiments. RR, CA, PG, and CG performed cell culture experiments. VB, ND, MHS, and MS helped with most experiments and analysis. S Giraudier, JS, and JJK collected essential patients’ specimens. CC and BC supervised the work and wrote the manuscript. S Ganesan, SAT, FG, NM, RR, CA, PG, CG, JS, OR, VB, ND, CL, MHS, MS, S Giraudier, JJK, CC, and BC read and edited the manuscript.

## Supplementary Material

Supplemental data

Unedited blot and gel images

Supporting data values

## Version 1 10/29/2024

In-Press Preview

## Version 2 12/06/2024

Electronic publication
