# Supplementary material for: Comprehensive analysis of mesenchymal cells reveals a dysregulated TGF-β/WNT/HOXB7 axis in patients with myelofibrosis
Source: JCI Insight. 2024 Dec 6;9(23):e173665. doi: 10.1172/jci.insight.173665 (PMC11623938; doi:10.1172/jci.insight.173665)
Supplement: Supplemental data [file jciinsight-9-173665-s198.pdf]

Comprehensive analysis of mesenchymal cells reveals a deregulated TGF-β/Wnt/HOXB7 axis in patients with myelofibrosis

Saravanan Ganesan[1], Sarah Awan-Toor[1], Fabien Guidez[1,2], Nabih Maslah[1,3], Laurence Rahimy[4], Céline Aoun[1], Panhong Gou[1], Chloé Guiguen[1], Juliette Soret[5], Odonchimeg Ravdan[3], Valeria Bisio[6], Nicolas Dulphy[6,7], Camille Lobry[8], Marie-Hélène Schlageter[3], Michèle Souyri[1], Stéphane Giraudier[1,3], Jean-Jacques Kiladjian[1,5], Christine Chomienne[1] and Bruno Cassinat [1,3]

Affiliation :

[1] INSERM UMRS 1131, Institut de Recherche Saint-Louis, Université Paris Cité, Paris F-75010, France
[2] INSERM U1232/LNC, Team Epi2THM, UBFC, Dijon, France
[3]AP-HP, Hôpital Saint-Louis, Service de Biologie Cellulaire, Paris F-75010, France
[4] Centre Hospitalier Région de Saint Omer, Service de Gériatrie, Helfaut F-62570, France
[5] Université Paris Cité, APHP, Hôpital Saint-Louis, Centre d'Investigations Cliniques, INSERM CIC 1427,
Paris F-75010, France
[6] INSERM U1160, Institut de Recherche Saint-Louis, Université Paris-Cité, Paris F-75010, France
[7]AP-HP, Hôpital Saint-Louis, Laboratoire d'Immunologie et d'Histocompatibilite, Paris F-75010, France
[8] INSERM U944, CNRS UMR7212, Institut de Recherche Saint Louis, Université Paris-Cité, Paris F-75010, France

Running Title: HOXB7 drives fibrosis in MPN

Supplementary Information

Supplementary Table 1: Patients Information

ID	Age	Sex	Initial Diagnosis	MF grade	Mutation	VAF	Cytoreductive treatment history	Driver Mutation in stroma
F-MPN1	77	F	ET	MF grade 2	JAK2 V617F	95	Hydroxyurea, Xagrid, Interferon	no
F-MPN2	36	M	ET	MF (prefibrotic stage)	CALR E364Rfs*49 (non type 1 del 52)	44	None	no
F-MPN3	58	M	ET	MF grade 2	MPL p.W515K	42	None	no
F-MPN4	32	F	ET	MF grade 1	CALR type 2	1	Interferon	no
F-MPN5	71	F	ET	MF grade 2	CALR del52 type 1	46	hydroxyurea, Interferon	no
F-MPN6	28	F	ET	MF grade 1	CALR ins5 type 2	40	Interferon	no
F-MPN7	19	F	PMF	PMF	CALR p.K368fs*46 type 1	38	Hydroxyurea	no
F-MPN8	64	F	ET	MF grade 3	CALR p.L367Tfs*46 type 1	44	None	no
F-MPN9	66	F	ET	MF grade 2	JAK2 V617F	59	Hydroxyurea, Interferon, Xagrid	no
F-MPN10	79	M	MF	Fibrosis grade 1	JAK2 V617F	3	Interferon, Ruxolitinib	no
F-MPN11	64	F	ET	MF grade 3	JAK2 V617F	64	None	no
F-MPN12	60	M	MF	MF grade 2	CALR	43	Hydroxyurea	no

Primer Sequences:

Oligonucleotides	Sequence
HOXB7 F	CGAGTTCCTTCAACATGCACT
HOXB7 R	TTTGCGGTCAGTTCCTGAGC
HOXB2 F	CGCCAGGATTCACCTTTCCTT
HOXB2 R	CCCTGTAGGCTAGGGGAGAG
HOXB5 F	AACTCCTTCTCGGGGCGTTAT
HOXB5 R	CATCCCATTGTAATTGTAGCCGT
HOXB9 F	TAATCAAAGACCCGGCTACG
HOXB9 R	CTACGGTCCCTGGTGAGGTA
HOXB-AS3 F	ACAACCGAGTGGAACTGACG
HOXB-AS3 R	AAGCCAAGGCTGTTCAAGGT
ACTA2 F	AAAAGACAGCTACGTGGGTGA
ACTA2 R	GCCATGTTCTATCGGGTACTTC
ACTB F	CCCTAAGGCCAACCGTGAA
ACTB R	CCAGAGGCATACAGGGACAAC
PPARG F	GCTGGCCTCCTTGATGAATA
PPARG R	TTGGGCTCCATAAAGTCACC
SOX9 F	GTACCCGCACTTGCACAAC
SOX9 R	TCTCGCTCTCGTTCAGAAGTC
PLZF1 F	AACCACAAGGCTGACGCTGTA
PLZF1 R	CATAGGTGCTGAAGTCCATGGA

Supplementary Figure 1: Representative flow cytometry histogram of markers used to characterize the expanded stromal cells.

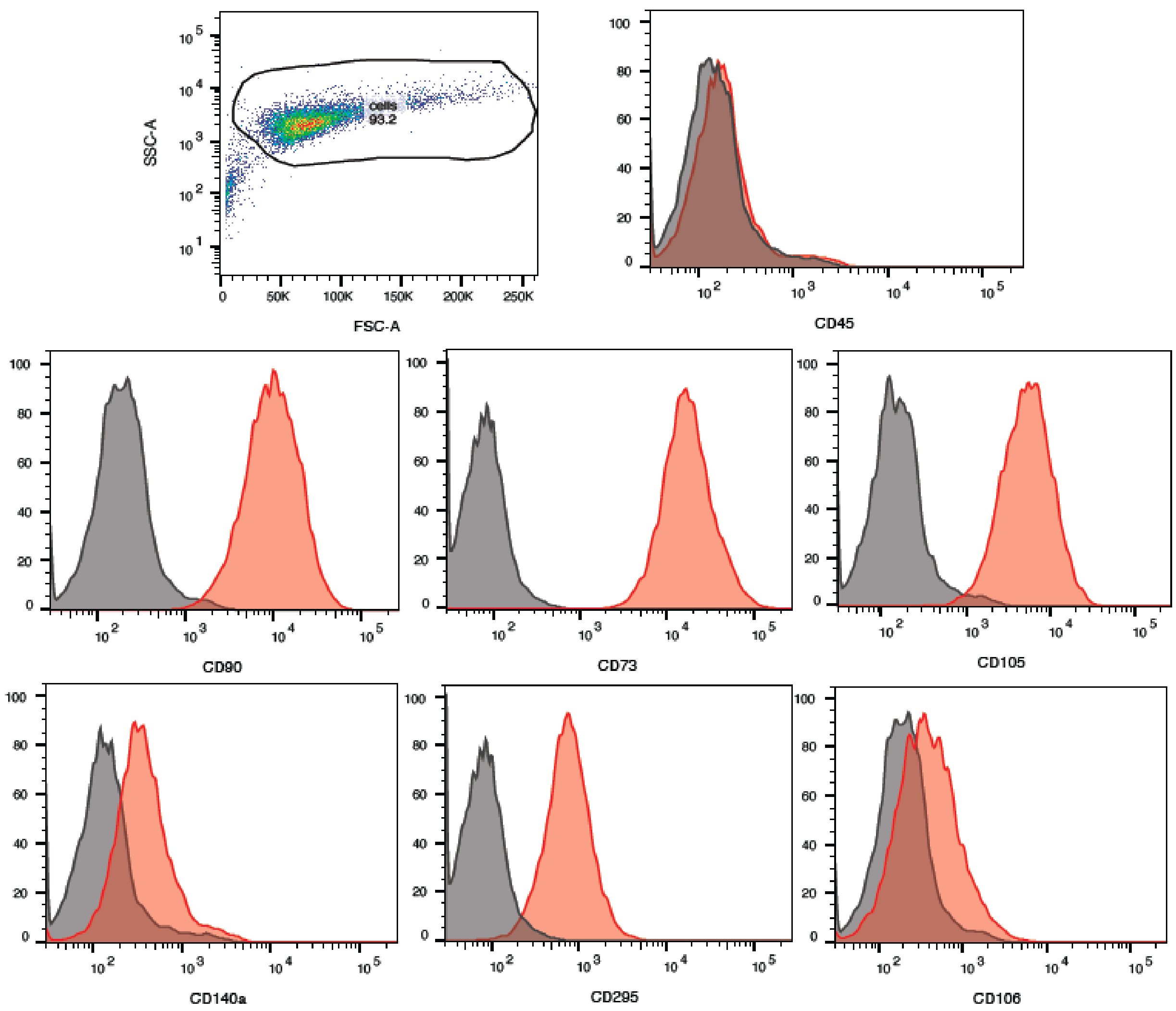

Supplementary Figure 2: Enrichment of osteoblast differentiation associated transcription factor motifs in F-MPN MSCs in comparison to control MSCs.

Rank	Motif	Name	P-value	log P-pvalue	q-value (Benjamini)	# Target Sequences with Motif	% of Targets Sequences with Motif
1	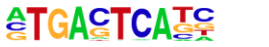	AP-1(bZIP)/ThioMac-PU.1-ChIP-Seq(GSE21512)/Homer	1e-279	-6.433e+02	0.0000	670.0	37.12%
2	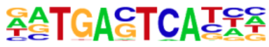	Atf3(bZIP)/GBM-ATF3-ChIP-Seq(GSE33912)/Homer	1e-269	-6.213e+02	0.0000	620.0	34.35%
3	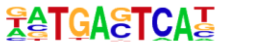	BATF(bZIP)/Th17-BATF-ChIP-Seq(GSE39756)/Homer	1e-268	-6.180e+02	0.0000	609.0	33.74%
4	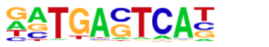	JunB(bZIP)/DendriticCells-Junb-ChIP-Seq(GSE36099)/Homer	1e-265	-6.111e+02	0.0000	568.0	31.47%
5	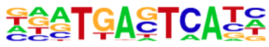	Fra1(bZIP)/BT549-Fra1-ChIP-Seq(GSE46166)/Homer	1e-263	-6.075e+02	0.0000	566.0	31.36%
6	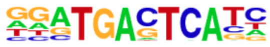	Fra2(bZIP)/Striatum-Fra2-ChIP-Seq(GSE43429)/Homer	1e-244	-5.623e+02	0.0000	513.0	28.42%
7	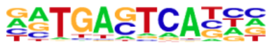	Fosl2(bZIP)/3T3L1-Fosl2-ChIP-Seq(GSE56872)/Homer	1e-215	-4.955e+02	0.0000	413.0	22.88%
8	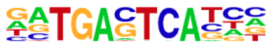	Jun-AP1(bZIP)/K562-cJun-ChIP-Seq(GSE31477)/Homer	1e-166	-3.839e+02	0.0000	307.0	17.01%
9	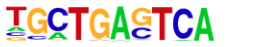	Bach2(bZIP)/OCILy7-Bach2-ChIP-Seq(GSE44420)/Homer	1e-87	-2.014e+02	0.0000	200.0	11.08%
10	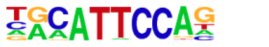	TEAD3(TEA)/HepG2-TEAD3-ChIP-Seq(Encode)/Homer	1e-60	-1.388e+02	0.0000	425.0	23.55%
11	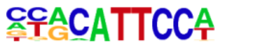	TEAD1(TEAD)/HepG2-TEAD1-ChIP-Seq(Encode)/Homer	1e-56	-1.308e+02	0.0000	377.0	20.89%
12	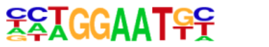	TEAD(TEA)/Fibroblast-PU.1-ChIP-Seq(Unpublished)/Homer	1e-47	-1.090e+02	0.0000	284.0	15.73%
13	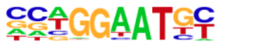	TEAD4(TEA)/Tropoblast-Tead4-ChIP-Seq(GSE37350)/Homer	1e-46	-1.064e+02	0.0000	327.0	18.12%

Supplementary Figure 3: Enrichment of adipocyte differentiation associated transcription factor (CEBPA) motifs in control MSCs in comparison to F-MPN MSCs.

Rank	Motif	Name	P-value	log P-pvalue	q-value (Benjamini)	# Target Sequences with Motif	% of Targets Sequences with Motif
8	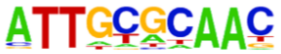	CEBP(bZIP)/ThioMac-CEBPb-ChIP-Seq(GSE21512)/Homer	1e-123	-2.845e+02	0.0000	426.0	20.77%

Supplementary Figure 4: Expression of HOXB7 (target) and HOXB9 (off-target) on HS-5 cells quantified by qRT-PCR after transduction with 2 different shRNAs, validating the use of the KD3 shRNA.

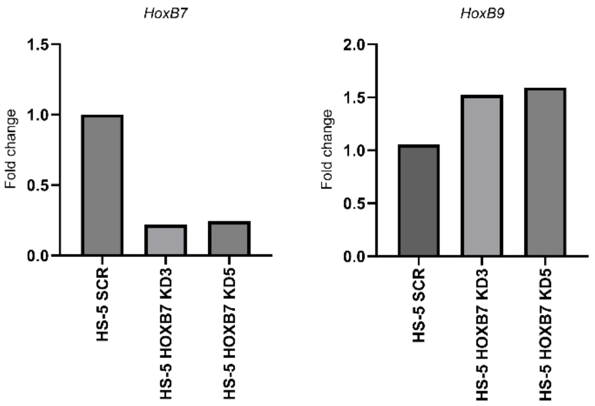

Supplementary Figure 5: Expression of HOXB7 in the presence of TGF-β (10ng/ml) and the JAK1/2 inhibitor ruxolitinib (Ruxo) measured by western blot. The expression is not altered even in the presence of ruxolitinib.

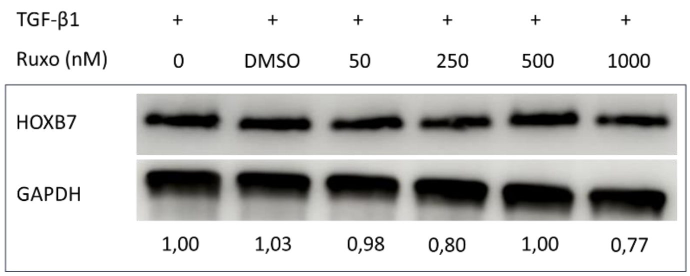

Supplementary Figure 6 : Antibodies used in this study

ANTIBODIES		
	Source	Reference
β-actin	Cell Signaling Technology	4970
HOXB7	Protein tech	12616-1-AP
a-SMA	Cell signaling/Santa Cruz	29245s/sc-53142
B-Catenin	Cell signaling/Santa Cruz	8480S/sc-7963
H3K9me3	Diagenode	C15410056
Tubulin	Santa Cruz	sc-5274
GAPDH	Santa Cruz	sc-32233
Cyclin D1	Protein tech	26939-1-AP
C-MYC	Santa Cruz	sc-40
Anti-Rabbit IgG HRP	Cell Signaling Technology	7074
Anti-Mouse IgG HRP	Invitrogen	G21046
Anti-Mouse IgG FITC	Invitrogen	31569
CD105	BD biosciences	560839
CD90	BD biosciences	561557
CD140a	BD biosciences	562799
CD295	BD biosciences	564376
CD45	BD biosciences	555482
CD106	BD biosciences	551146
CD73	BD biosciences	560847
