# Supplementary figures and images for: Comprehensive analysis of mesenchymal cells reveals a dysregulated TGF-β/WNT/HOXB7 axis in patients with myelofibrosis
Source: JCI Insight. 2024 Dec 6;9(23):e173665. doi: 10.1172/jci.insight.173665 (PMC11623938; doi:10.1172/jci.insight.173665)

# Figure 4b

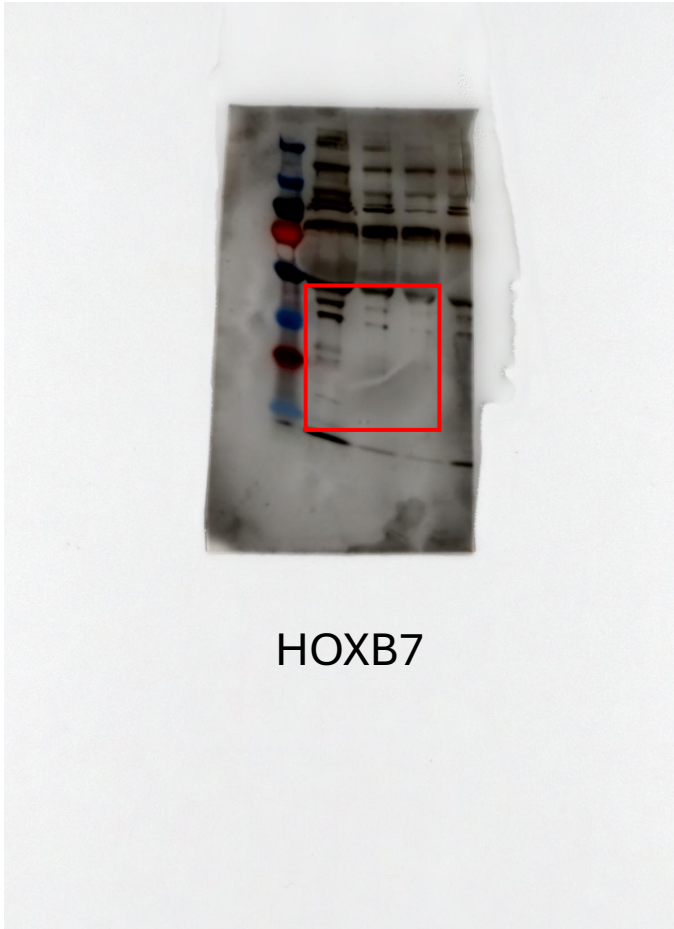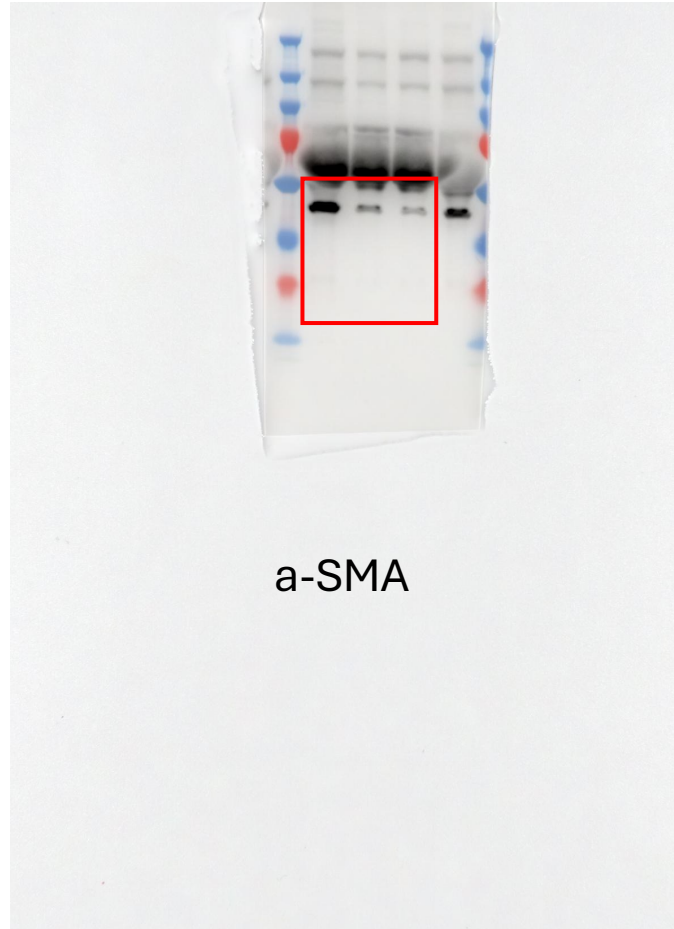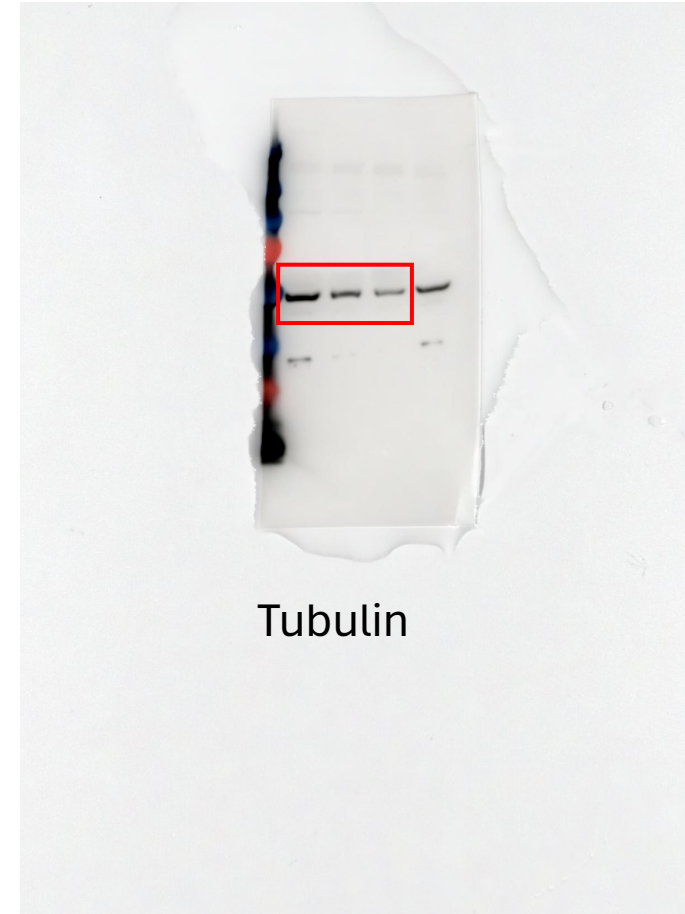

# Figure 5a

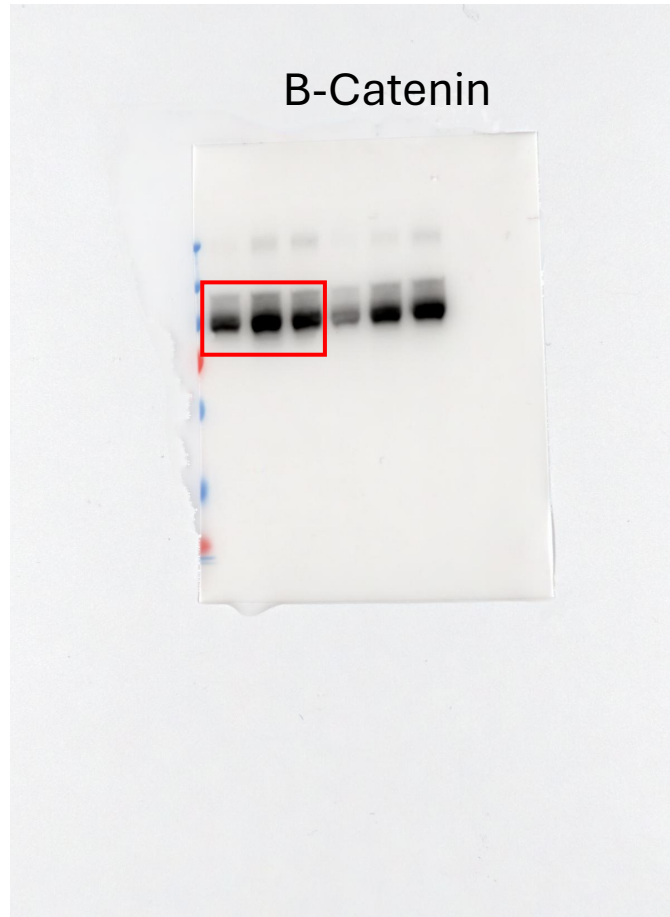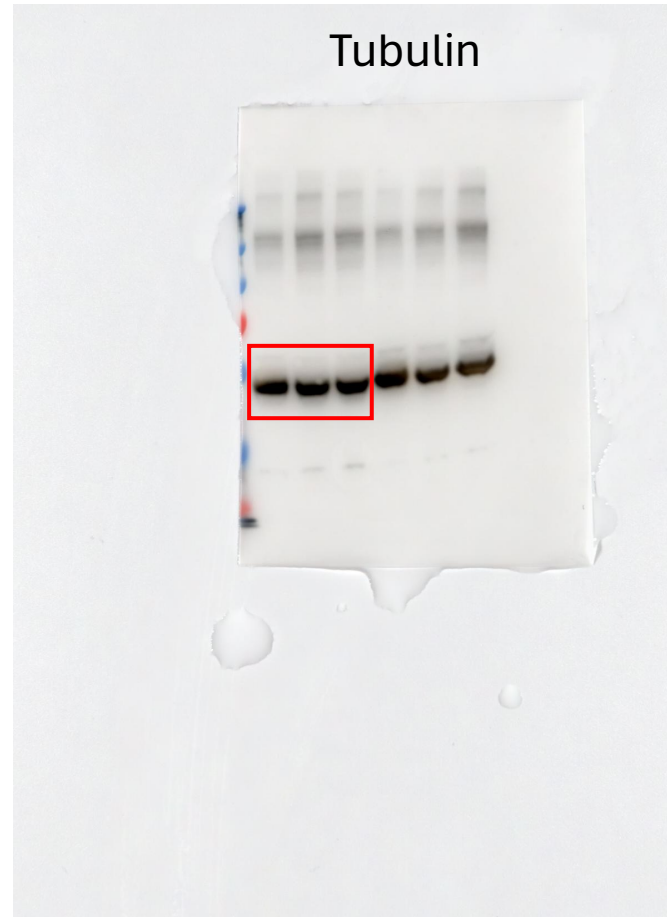

# Figure 5d

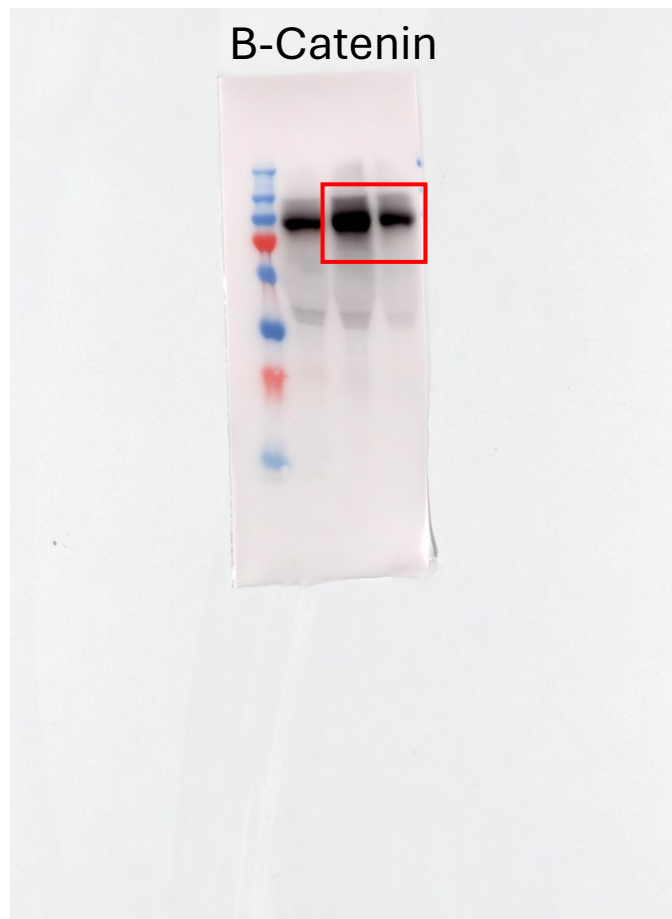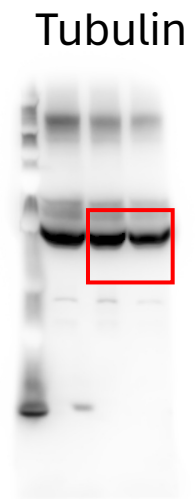

Figure 5d

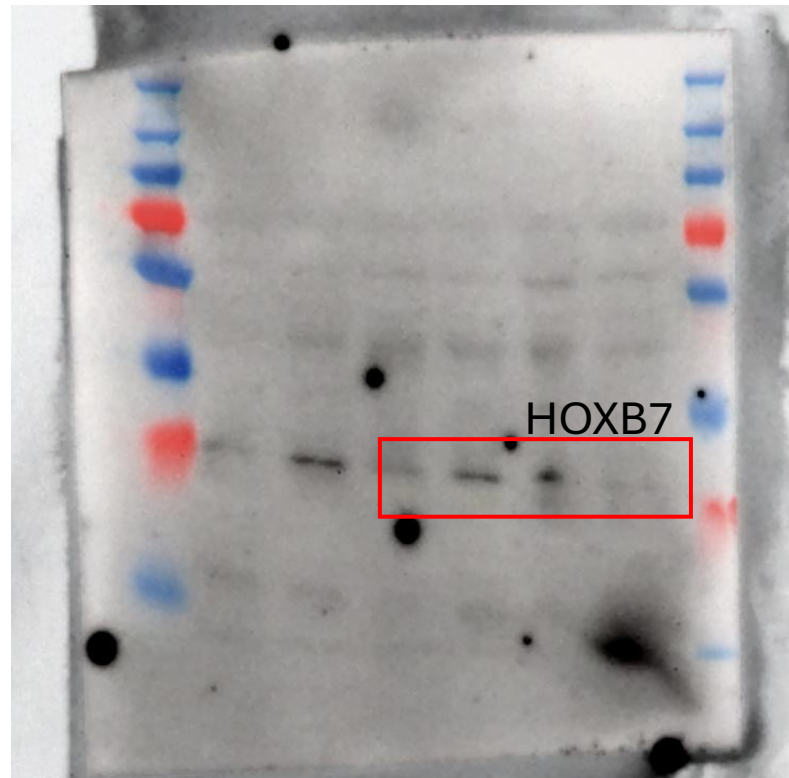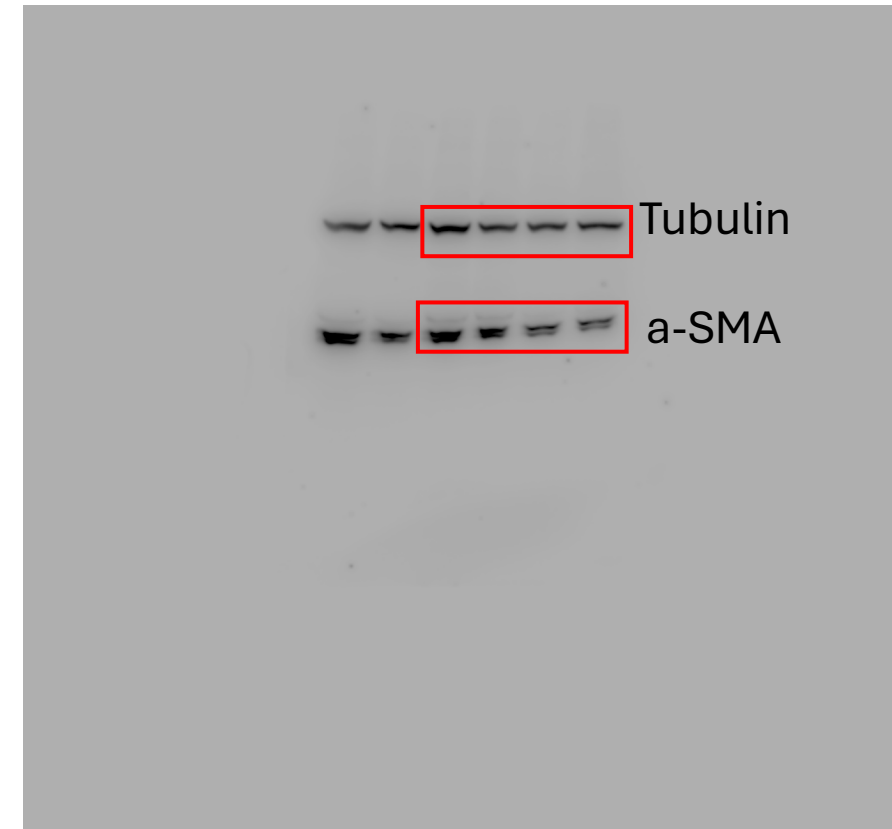

Figure 5F

HOX B7

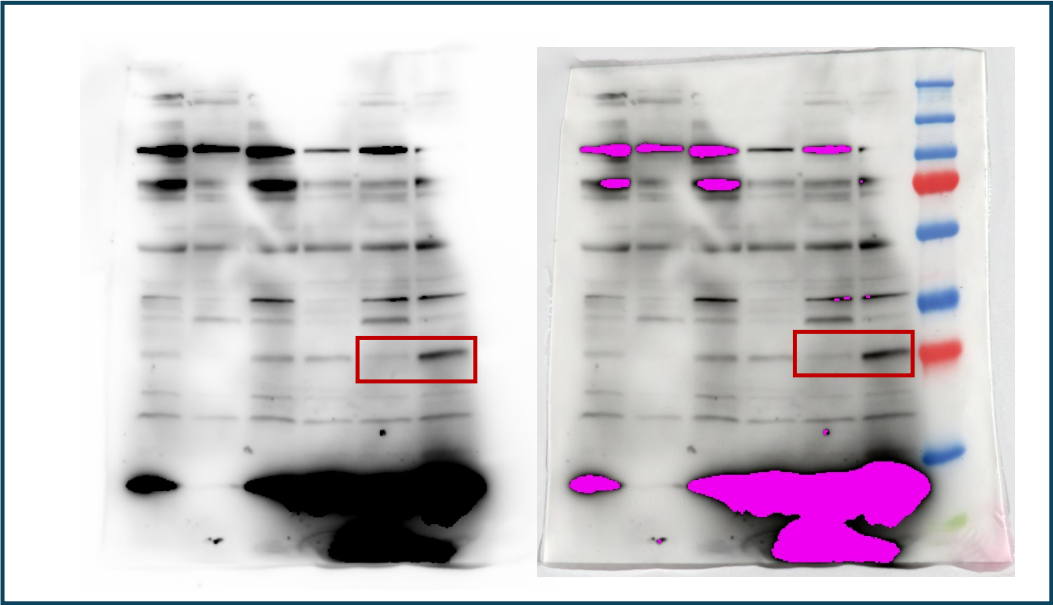

SMA

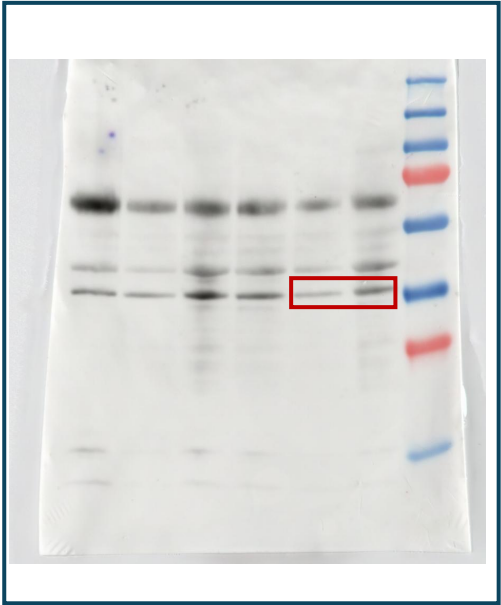

Cyclin D1

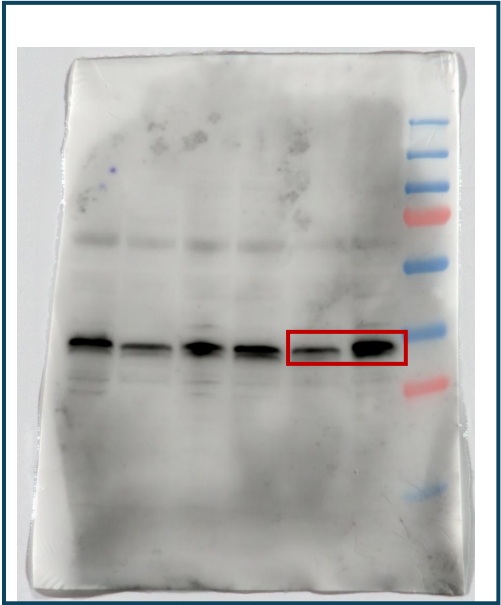

C-myc

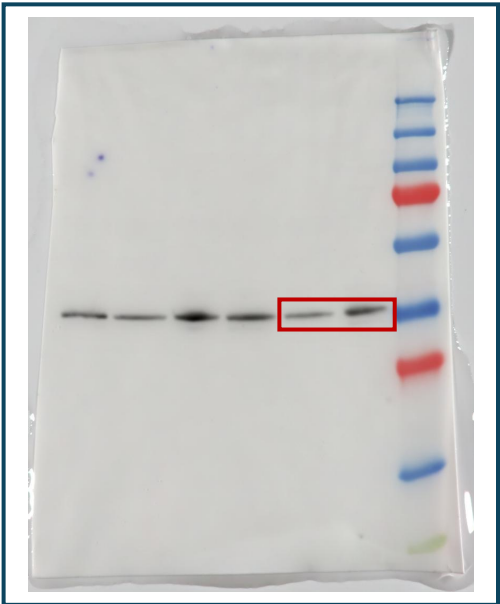

GAPDH

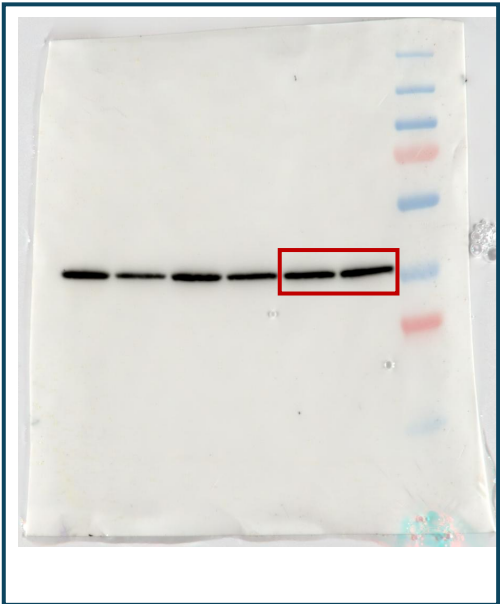

HOXB7

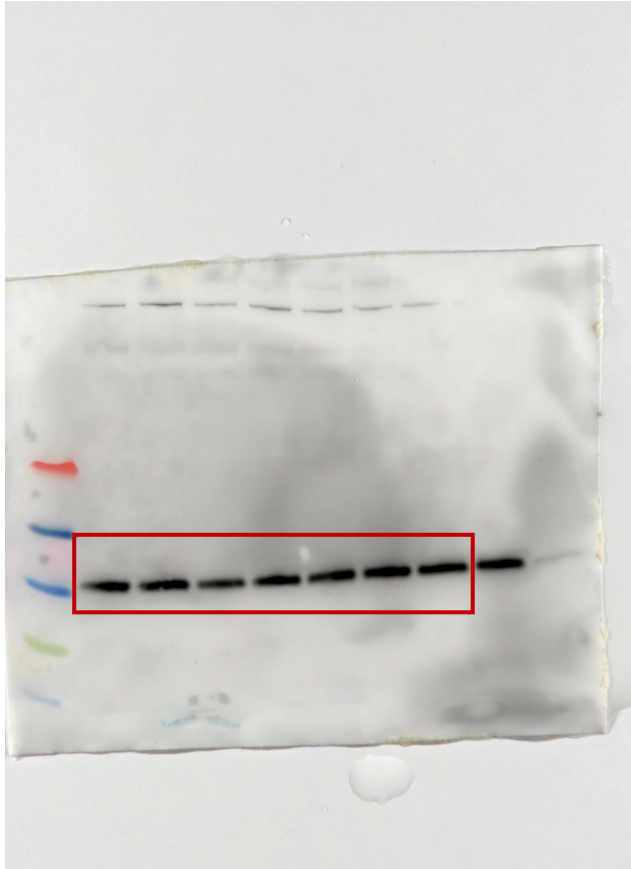

GAPDH

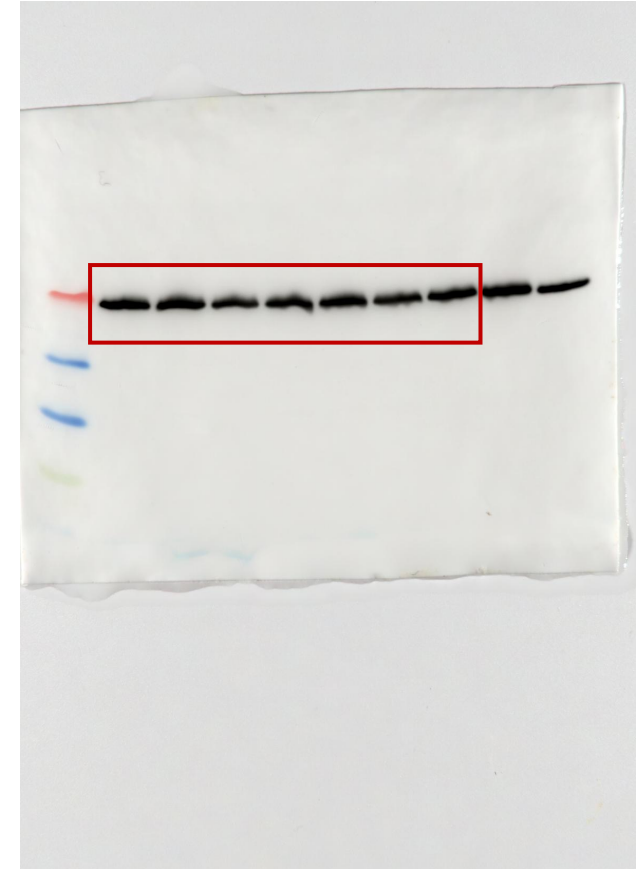

Supplement: Unedited blot and gel images [file jciinsight-9-173665-s199.pdf]
